# Cancer Risk According to Alcohol Consumption Trajectories: A Population-based Cohort Study of 2.8 Million Korean Men

**DOI:** 10.2188/jea.JE20220175

**Published:** 2023-12-05

**Authors:** Thi Tra Bui, Minji Han, Ngoc Minh Luu, Thi Phuong Thao Tran, Min Kyung Lim, Jin-Kyoung Oh

**Affiliations:** 1 Department of Cancer Control and Population Health, National Cancer Center Graduate School of Cancer Science and Policy, Goyang, Republic of Korea; 2 Division of Cancer Prevention, National Cancer Control Institute, National Cancer Center, Goyang, Republic of Korea; 3 Department of Social and Preventive Medicine, College of Medicine, Inha University, Incheon, Republic of Korea

**Keywords:** alcohol, cancer, cohort, Korea, trajectory

## Abstract

**Background:**

Alcohol drinking behaviors change temporally and can lead to changes in related cancer risks; previous studies have been unable to identify the association between the two using a single-measurement approach. Thus, this study aimed to investigate the association of drinking trajectories with the cancer risk in Korean men.

**Methods:**

A trajectory analysis using group-based trajectory modeling was performed on 2,839,332 men using data on alcohol drinking levels collected thrice during the Korean National Health Insurance Service’s general health screening program conducted between 2002 and 2007. Cox proportional hazards regression was performed to evaluate the associations between drinking trajectories and cancer incidence, after adjustments for age, income, body mass index, smoking status, physical activity, family history of cancer, and comorbidities.

**Results:**

During 10.5 years of follow-up, 189,617 cancer cases were recorded. Six trajectories were determined: non-drinking, light, moderate, decreasing-heavy, increasing-heavy, and steady-heavy. Light-to-heavy alcohol consumption increased the risk for all cancers combined in a dose-dependent manner (adjusted hazard ratio [aHR] 1.03; 95% confidence interval [CI], 1.02–1.05 for light drinking, aHR 1.06; 95% CI 1.05–1.08 for moderate drinking, aHR 1.19; 95% CI, 1.16–1.22 for decreasing-heavy drinking, aHR 1.23; 95% CI, 1.20–1.26 for increasing-heavy drinking, and aHR 1.33; 95% CI, 1.29–1.38 for steady-heavy drinking [*P*-trend <0.001]). Light-to-heavy alcohol consumption was linked to lip, oral cavity, pharyngeal, esophageal, colorectal, laryngeal, stomach, and gallbladder and biliary tract cancer risks, while heavy alcohol consumption was associated with hepatic, pancreatic, and lung cancer risks. An inverse association was observed for thyroid cancer. The cancer risks were lower for decreasing-heavy drinkers, compared to steady-heavy drinkers.

**Conclusion:**

No safe drinking limits were identified for cancer risks; reduction in heavy intake had protective effects.

## INTRODUCTION

Alcoholic beverages are classified as Group 1 carcinogens, with sufficient evidence supporting their role in cavity, pharyngeal, laryngeal, esophageal, colorectal, hepatic, and female breast cancers.^1^ Positive associations have been suggested between alcoholic beverages and gastric, pancreatic, gallbladder, lung, and prostate cancers.^2^^,^^3^ Some national guidelines recommend moderate drinking due to the protective effects of light-to-moderate drinking on the risk of cardiovascular diseases. However, whether such safe drinking limits exist for the risk of cancer remains controversial.^4^^,^^5^ In previous studies, estimation of an association between alcohol consumption and cancer risk mostly relied on single measurements of alcohol intake.^2^^,^^3^ However, temporal changes in the drinking amount have been reported.^6^^,^^7^ One study revealed that the alcohol consumption-associated cancer risk differed at different times.^8^ Thus, temporally different drinking patterns may lead to changes in the related cancer risks. Associations between alcohol consumption and cancer risk were reversible following drinking cessation.^9^ However, this finding is derived from retrospective studies, some of which analyzed subsets of former drinkers, which are not representative of the general population.^9^ Further studies on alcohol consumption and its health effects must involve a broader population and perform repeated measurements to reveal alcohol drinking patterns with greater accuracy.^3^

Recent epidemiological studies have adopted trajectory analyses^10^^,^^11^ to identify behavioral patterns over time. Several studies have investigated alcohol consumption trajectories^7^^,^^12^^,^^13^ and their associations with health outcomes, including all-cause mortality,^14^ cardiovascular disease incidence^15^ and mortality,^14^ and cancer mortality.^14^ However, there is limited research on alcohol consumption trajectories and cancer development.^6^^,^^16^ A recent Australian cohort study examined lifetime drinking patterns and suggested critical timelines regarding alcohol-related cancer etiology and prevention.^6^ However, it examined a limited number of cancers and focused on alcohol-related cancers.^6^ Therefore, using a trajectory analysis to comprehensively investigate the cancer risk based on alcohol drinking patterns would be meaningful. This population-based cohort study aimed to evaluate the associations of alcohol consumption trajectories with the risk of 20 cancers in Korean men.

## METHODS

### Data source and study population

The National Health Information Database of the National Health Insurance Service (NHIS) is a public database covering the entire Korean population (50 million).^17^^,^^18^ The database comprises annually collected data on socio-demographics, healthcare utilization, mortality, and health examinations performed periodically.^17^ Insured adults and their dependents are eligible for a biennial general health examination.^18^

**Figure 1.**
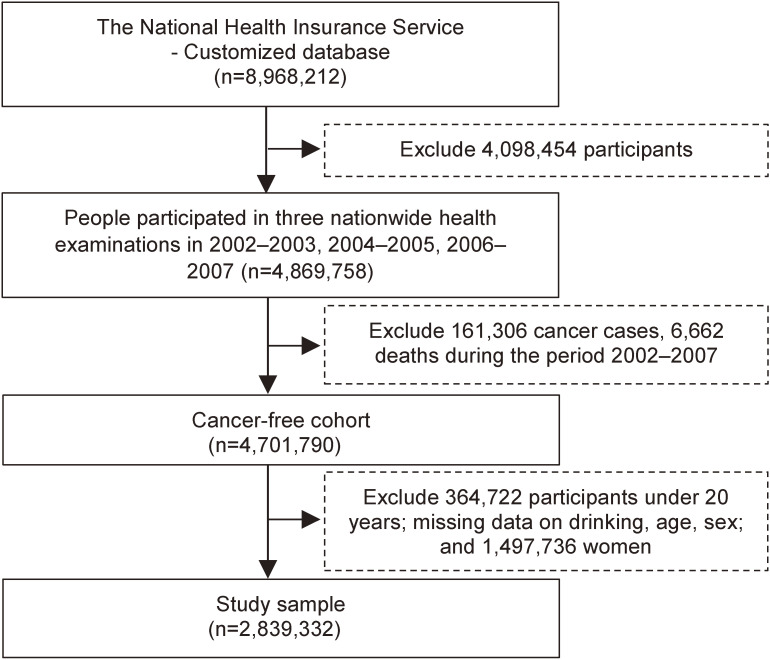
Study flowchart

We referred to a customized NHIS database of 8,968,212 participants who underwent the 2002–2003 nationwide health examination and were followed up through to 2018. Among these, 4,869,758 individuals who underwent the 2002–2003, 2004–2005, and 2006–2007 nationwide health examinations were included. However, 161,306 individuals diagnosed with cancer before 2008; 6,662 individuals who died before 2008; 364,722 individuals under 20 years of age or with missing baseline data on age, sex, or alcohol consumption at the three aforementioned examinations; and 1,497,736 women were excluded. The remaining 2,839,332 men were finally included in this study. The study flow is shown in Figure 1.

This study was exempted from review by the Institutional Review Board of the National Cancer Center, Korea (NCC2018-0279), because it used anonymized secondary data. The requirement of informed consent was waived for the same reason.

### Exposure and covariates

#### Alcohol consumption

Data on the drinking frequency and amount (per time) were collected using a self-administered questionnaire. Soju is the most commonly consumed alcoholic beverage in Korea; thus, the drinking amount per time was expressed in terms of soju bottles. Alcohol consumption (g/day) was estimated using the following formula:

([Q×V×C×d]×F)/28

where Q, V, C, d, and F represent the drinking amount per time (bottles), volume of the soju bottle (360 mL), percentage of ethanol by volume (20%), specific gravity of ethanol (0.8 g/mL), and monthly drinking frequency (reported for 28 days), respectively. Alcohol consumption was then categorized into six levels: 0, 1–9.9, 10–19.9, 20–29.9, 30–49.9, and ≥50 g/day. Data on the drinking levels collected during the three examinations were used for a trajectory analysis to determine the drinking patterns during 2002–2007. For people attending two examinations within a wave, data from the earlier measurement were used.

#### Covariates

Baseline data (2002–2003) on the age, income, body mass index (BMI), smoking status, physical activity, family history of cancer, and Charlson Comorbidity Index (CCI) were used to adjust for confounding variables. Age and CCI^19^ were considered continuous, while categorical covariates included income (quintiles), BMI^20^ (<18.5, 18.5–22.9, 23.0–24.9, and ≥25.0 kg/m^2^), smoking status (never, former, and current smokers), physical activity (0, 1–2, 3–4, 5–6, and 7 times/week), and family history of cancer (yes or no).

### Case ascertainment

Cancer cases were defined by primary cancer diagnosis in hospitals, using the 10^th^ revision of the International Classification of Diseases (ICD-10) codes. Participants diagnosed before 2008 were excluded from the study population to form a cancer-free cohort. To obtain conservative results, cancer cases ascertained during 2008–2018 were confirmed by “V193,” a special code introduced by the NHIS from September 2005 to expand the insurance benefits for patients with cancer. The cancer date was defined as the first date of primary cancer diagnosis. The outcomes of interest included all cancers combined and alcohol-related cancers, which were categorized into the alcohol-related cancer group, including lip, oral cavity, pharyngeal, esophageal, colorectal, hepatic, and laryngeal cancers.^1^ All cancers comprised these alcohol-related cancers and 15 other cancers (according to the GLOBOCAN cancer dictionary; hereafter referred to as “other cancer types”) for which evidence on a causal relationship with alcohol consumption is lacking.^21^

### Statistical methods

#### Trajectory analysis

Trajectory analysis is used to determine distinct subgroups of similar behavioral patterns over time in a defined population.^10^ We adopted group-based trajectory modelling to investigate alcohol consumption, using the PROC TRAJ procedure in SAS 9.4 (SAS Institute Inc., Cary, NC, USA).^11^ Among the different trajectory analysis strategies, this semi-parametric strategy is commonly used in epidemiological research because it produces less complex and easy-to-interpret models that require less computing time.^10^

First, during group number selection, one-to-seven-group quadratic models were examined, given that up to six alcohol consumption trajectories were identified in previous studies.^7^^,^^12^^,^^13^ The optimal number of groups was identified based on the Bayesian information criterion (BIC).^11^ However, the BIC values increased as more groups were added to the models (eTable 1). Then, a decision was taken based on group membership (≥1%), model parsimony, and distinct model features (eTable 2). A six-group model was selected because of the ability to separate abstainers and light drinkers and to subgroup different patterns of heavy alcohol consumption.

**Figure 2.**
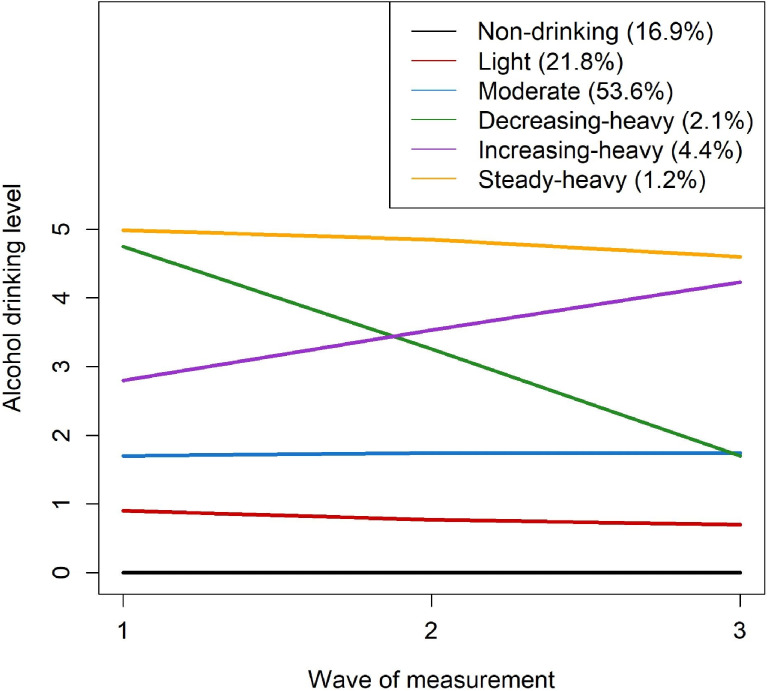
Alcohol consumption trajectory model

Next, during group order optimization, groups with a non-significant quadratic order (*P*-value <0.05) were reduced to those with a linear order.^22^ The never-drinker group was set at zero order. Models with extreme standard errors in the parameters were excluded. Based on the BIC values, we selected the six-group model with zero, linear, linear, linear, linear, and linear orders (Figure 2). Finally, the following six alcohol consumption trajectories were identified: 1) TR1, non-drinking (group membership after assignment: *N* = 480,832, 16.9%; drinking amount over three waves of measurement: mean 0; standard deviation [SD], 0 g/day); 2) TR2, light (*N* = 619,617, 21.8%; mean 3.2; SD, 2.1 g/day); 3) TR3, moderate (*N* = 1,521,465, 53.6%; mean 12.3; SD, 6.5 g/day); 4) TR4, decreasing-heavy (*N* = 60,511, 2.1%; mean 35.2; SD, 9.1 g/day); 5) TR5, increasing-heavy (*N* = 123,122, 4.4%; mean 38.7; SD, 9.3 g/day); and 6) TR6, steady-heavy (*N* = 33,785; 1.2%; mean 60.9; SD, 13.9 g/day).

The final model was then evaluated using the average posterior probability (AvePP) of assignment and the odds of correct classification (OCC). An AvePP ≥0.7 and an OCC ≥5.0 are recommended to indicate good group assignment.^11^ Almost all groups in the selected models had a high AvePP (≥0.8) and OCC (≥12.3), except for the TR3 (moderate) group (OCC = 4.5; eTable 3). However, considering its OCC value was close to the recommended value, TR3 was also accepted for further analysis.

#### Cox proportional hazard regression

The Cox proportional hazard method was used to evaluate the associations between alcohol consumption and cancer incidence. The group assignment to alcohol consumption trajectories was treated as an independent variable (categorical). Furthermore, the baseline drinking level was utilized for comparison with the trajectory approach. Censored cases included participants who died during 2008–2018 or did not experience any events by the end of 2018. The time-to-event (years) was defined as the duration from January 1, 2008, to the cancer date, censored date, or December 31, 2018, whichever occurred first. It was assumed that a patient diagnosed with a specific cancer type was no longer at risk of other malignancies; thus, they were treated as censored on their cancer date in the analysis of other cancers. The non-drinking group was used as the reference.

#### Sensitivity analysis

Several sensitivity analyses were performed. To elicit the implications for behavioral change, the association of alcohol trajectories with the cancer risk was evaluated with TR6 (steady-heavy) as the reference. Because smoking status is highly correlated with drinking status, a subgroup analysis was performed for non-smokers. During the consideration of latent cases, we excluded cancer cases diagnosed in the year after 2007 from the study population. Furthermore, given that each trajectory of heavy drinkers, namely TR4 (decreasing-heavy), TR5 (increasing-heavy), and TR6 (steady-heavy), accounted for less than 5% of the study population, we combined these three groups into one (“heavy drinkers”) and examined the association of alcohol trajectories with the cancer risk.

## RESULTS

### General characteristics of the study population and cancer incidence

The general characteristics of the study population (by the alcohol drinking trajectories) are presented in Table 1 (descriptions by the baseline alcohol drinking levels in eTable 4). At the baseline, the mean age of the participants was 41.5 (SD, 11.6) years. Participants with excess weight (BMI ≥23 kg/m^2^) accounted for 61.7% of the total cohort. Only 18.7% participants performed physical exercises regularly (≥3 times/week). Approximately 50% of the participants were current smokers; approximately 71.5% reported being alcohol drinkers at the baseline and 14.0% consumed ≥20 g/day of alcohol. The trajectory approach assigned alcohol drinking levels consistent with the single-measurement classifications. Moderate and heavy drinkers were more likely to be older than light drinkers.

**Table 1.** General characteristics of the study population at study entry (2002–2003)

	Total (*n* = 2,839,332)	(1) Non-drinking (*n* = 480,832)	(2) Light (*n* = 619,617)	(3) Moderate (*n* = 1,521,465)	(4) Decreasing-heavy (*n* = 60,511)	(5) Increasing-heavy (*n* = 123,122)	(6) Steady-heavy (*n* = 33,785)
**Age group, *N* (%)**							
20–<30 years	414,361 (14.6)	45,013 (9.4)	97,665 (15.8)	250,627 (16.5)	12,795 (10.4)	6,482 (10.7)	1,779 (5.3)
30–<40 years	956,856 (33.7)	123,736 (25.7)	209,055 (33.7)	566,077 (37.2)	36,342 (29.5)	15,037 (24.9)	6,609 (19.6)
40–<50 years	808,558 (28.5)	134,635 (28.0)	167,233 (27.0)	437,288 (28.7)	39,108 (31.8)	18,727 (30.9)	11,567 (34.2)
50–<60 years	403,361 (14.2)	92,814 (19.3)	87,257 (14.1)	182,621 (12.0)	21,058 (17.1)	11,397 (18.8)	8,214 (24.3)
60–<70 years	198,786 (7.0)	61,729 (12.8)	45,033 (7.3)	69,227 (4.6)	11,251 (9.1)	6,936 (11.5)	4,610 (13.6)
≥70 years	57,410 (2.0)	22,905 (4.8)	13,374 (2.2)	15,625 (1.0)	2,568 (2.1)	1,932 (3.2)	1,006 (3.0)

**Income**, ***N* (%)**							
1^st^ quintile	255,292 (9.0)	54,850 (11.4)	57,869 (9.3)	120,829 (7.9)	12,043 (9.8)	6,267 (10.4)	3,434 (10.2)
2^nd^ quintile	390,612 (13.8)	69,942 (14.5)	85,708 (13.8)	201,888 (13.3)	18,337 (14.9)	9,667 (16.0)	5,070 (15.0)
3^rd^ quintile	675,213 (23.8)	109,303 (22.7)	144,799 (23.4)	367,757 (24.2)	30,311 (24.6)	14,921 (24.7)	8,122 (24.0)
4^th^ quintile	700,008 (24.7)	111,855 (23.3)	149,416 (24.1)	385,606 (25.3)	30,502 (24.8)	14,283 (23.6)	8,346 (24.7)
5^th^ quintile	705,478 (24.8)	120,629 (25.1)	155,229 (25.1)	380,311 (25.0)	28,093 (22.8)	13,198 (21.8)	8,018 (23.7)
Missing	112,729 (4.0)	14,253 (3.0)	26,596 (4.3)	65,074 (4.3)	3,836 (3.1)	2,175 (3.6)	795 (2.4)

**Family history of cancer, *N* (%)**	367,548 (12.9)	60,887 (12.7)	73,402 (11.8)	202,716 (13.3)	8,338 (13.8)	17,016 (13.8)	5,189 (15.4)

**Body mass index, *N* (%)**							
<18.5 kg/m^2^	60,652 (2.1)	13,672 (2.8)	14,515 (2.3)	27,146 (1.8)	1,947 (1.6)	1,045 (1.7)	605 (1.8)
18.5–22.9 kg/m^2^	1,023,640 (36.1)	181,328 (37.7)	235,578 (38.0)	536,885 (35.3)	39,582 (32.1)	19,235 (31.8)	11,032 (32.7)
22.9–24.9 kg/m^2^	780,828 (27.5)	128,737 (26.8)	169,391 (27.3)	425,510 (28.0)	32,711 (26.6)	15,751 (26.0)	8,728 (25.8)
≥25 kg/m^2^	972,424 (34.2)	156,248 (32.5)	199,260 (32.2)	530,386 (34.9)	48,754 (39.6)	24,402 (40.3)	13,374 (39.6)
Missing	1,788 (0.1)	847 (0.2)	873 (0.1)	1,538 (0.1)	128 (0.1)	78 (0.1)	46 (0.1)

**Alcohol drinking, *N* (%)**							
0 g/day	810,493 (28.5)	480,832 (100)	247,558 (40.0)	76,925 (5.1)	5,178 (4.2)	0 (0)	0 (0)
1–9.9 g/day	891,728 (31.4)	0 (0)	291,129 (47.0)	590,674 (38.8)	9,925 (8.1)	0 (0)	0 (0)
10–19.9 g/day	738,441 (26.0)	0 (0)	70,383 (11.4)	637,200 (41.9)	30,858 (25.1)	0 (0)	0 (0)
20–29.9 g/day	227,533 (8.0)	0 (0)	8,608 (1.4)	175,108 (11.5)	43,817 (35.6)	0 (0)	0 (0)
30–49.9 g/day	63,085 (2.2)	0 (0)	1,939 (0.3)	32,436 (2.1)	21,274 (17.3)	7,436 (12.3)	0 (0)
≥50 g/day	108,052 (3.8)	0 (0)	0 (0)	9,122 (0.6)	12,070 (9.8)	53,075 (87.7)	33,785 (100)

**Smoking status,** *n* (%)							
Never smoker	1,014,304 (35.7)	269,547 (56.1)	270,465 (43.7)	427,420 (28.1)	27,151 (22.1)	12,790 (21.1)	6,931 (20.5)
Former smoker	445,732 (15.7)	56,795 (11.8)	94,035 (15.2)	261,963 (17.2)	18,989 (15.4)	8,867 (14.7)	5,083 (15.0)
Current smoker	1,367,240 (48.2)	152,571 (31.7)	251,396 (40.6)	826,422 (54.3)	76,471 (62.1)	38,684 (63.9)	21,696 (64.2)
Missing	12,056 (0.4)	1,919 (0.4)	3,721 (0.6)	5,660 (0.4)	511 (0.4)	170 (0.3)	75 (0.2)

**Physical exercise,** *n* (%)							
0 times/week	1,238,772 (43.6)	232,243 (48.3)	280,387 (45.3)	616,044 (40.5)	31,266 (51.7)	59,968 (48.7)	18,864 (55.8)
1–2 times/week	997,719 (35.1)	145,246 (30.2)	210,266 (33.9)	581,584 (38.2)	15,828 (26.2)	37,077 (30.1)	7,718 (22.8)
3–4 times/week	326,480 (11.5)	52,016 (10.8)	69,234 (11.2)	182,818 (12)	6,027 (10)	13,311 (10.8)	3,074 (9.1)
5–6 times/week	71,344 (2.5)	12,185 (2.5)	14,823 (2.4)	38,482 (2.5)	1,592 (2.6)	3,325 (2.7)	937 (2.8)
Almost everyday	132,090 (4.7)	28,570 (5.9)	27,951 (4.5)	61,839 (4.1)	4,540 (7.5)	6,572 (5.3)	2,618 (7.7)
Missing	72,927 (2.6)	10,572 (2.2)	16,956 (2.7)	40,698 (2.7)	1,258 (2.1)	2,869 (2.3)	574 (1.7)

**Charlson Comorbidity Index,** *n* (%)							
0	2,805,892 (98.8)	471,826 (98.1)	611,025 (98.6)	1,508,451 (99.1)	59,639 (98.6)	121,619 (98.8)	33,332 (98.7)
1	27,490 (1.0)	7,055 (1.5)	6,990 (1.1)	11,120 (0.7)	717 (1.2)	1,247 (1.0)	361 (1.1)
2	4,358 (0.2)	1,337 (0.3)	1,177 (0.2)	1,458 (0.1)	118 (0.2)	195 (0.2)	73 (0.2)
≥3	1,592 (0.1)	614 (0.1)	425 (0.1)	436 (0.1)	37 (0.1)	61 (0)	19 (0.1)

After a mean follow-up of 10.5 years, 189,617 cancer cases were recorded (Table 2). The leading cancers included gastric (19.8%), colorectal (14.2%), lung (11.3%), prostate (10.4%), liver (8.8%), and thyroid (8.4%) cancers.

**Table 2.** Number of cancer incident cases during 2008–2018

Cancer types	ICD-10	*N* (%)
Lip, oral cavity, and pharynx	C00–14	3,071 (1.6)
Esophagus	C15	2,652 (1.4)
Stomach	C16	37,614 (19.8)
Colon and rectum	C18–20	26,887 (14.2)
Liver	C22	16,624 (8.8)
Gallbladder and biliary tract	C23–24	4,266 (2.2)
Pancreas	C25	4,722 (2.5)
Larynx	C32	1,602 (0.8)
Lung	C33–34	21,464 (11.3)
Breast	C50	144 (0.1)
Prostate	C61	19,686 (10.4)
Testis	C62	251 (0.1)
Kidney	C64	5,270 (2.8)
Bladder	C67	6,332 (3.3)
Brain	C70–72	2,090 (1.1)
Thyroid gland	C73	15,901 (8.4)
Hodgkin lymphoma	C81	158 (0.1)
Non-Hodgkin lymphoma	C82–86,96	3,592 (1.9)
Multiple myeloma, malignant plasma cell neoplasms	C90	1,245 (0.7)
Leukemia	C91–95	2,556 (1.3)
Others	Others	13,490 (7.1)

Total	C00–97	189,617

ICD-10, the International Classification of Diseases, 10th Revision.

### Cancer risks according to alcohol consumption

**Figure 3.**
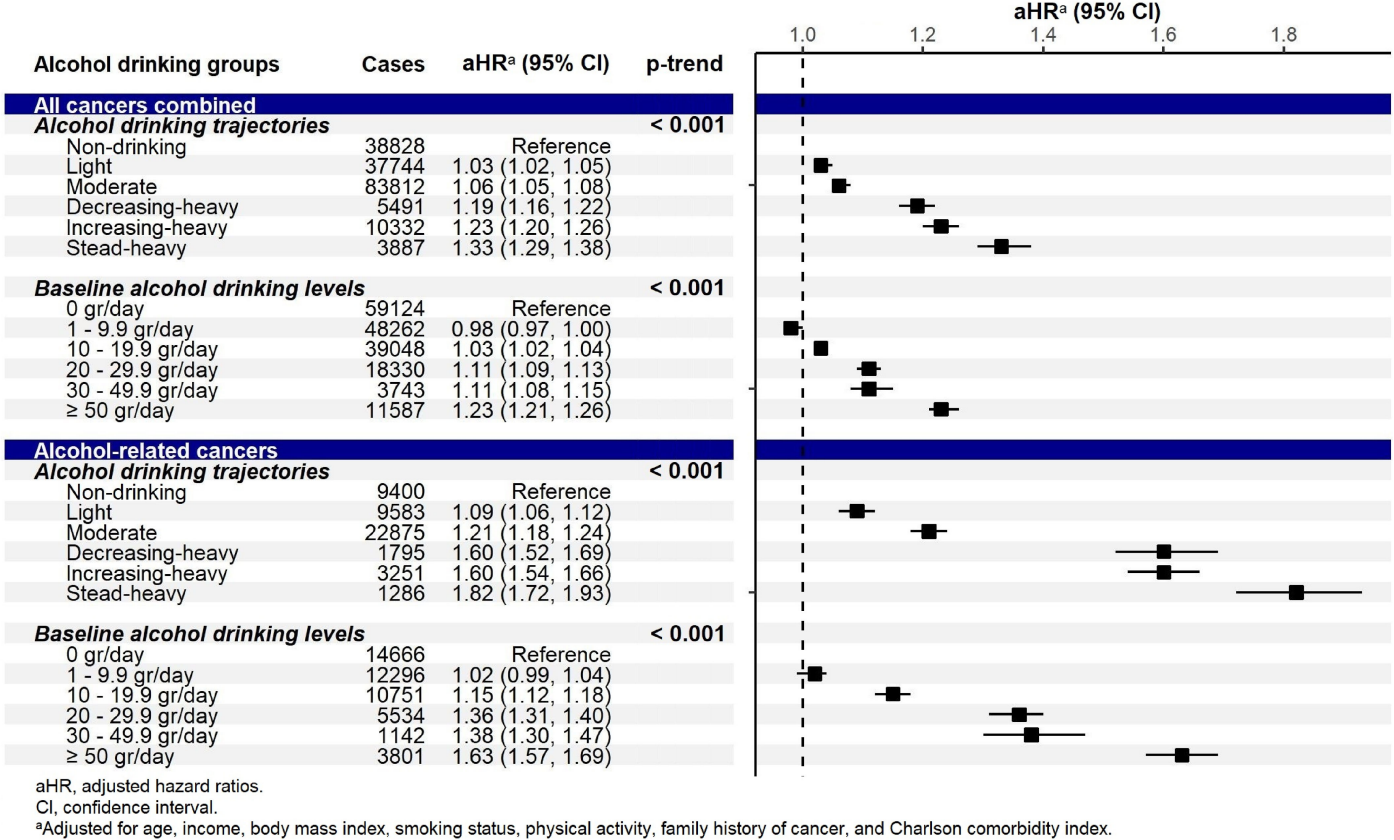
Adjusted hazard ratios (95% confidence interval) for the association between alcohol consumption and the risk for all cancers combined and for all alcohol-related cancers combined

**Figure 4.**
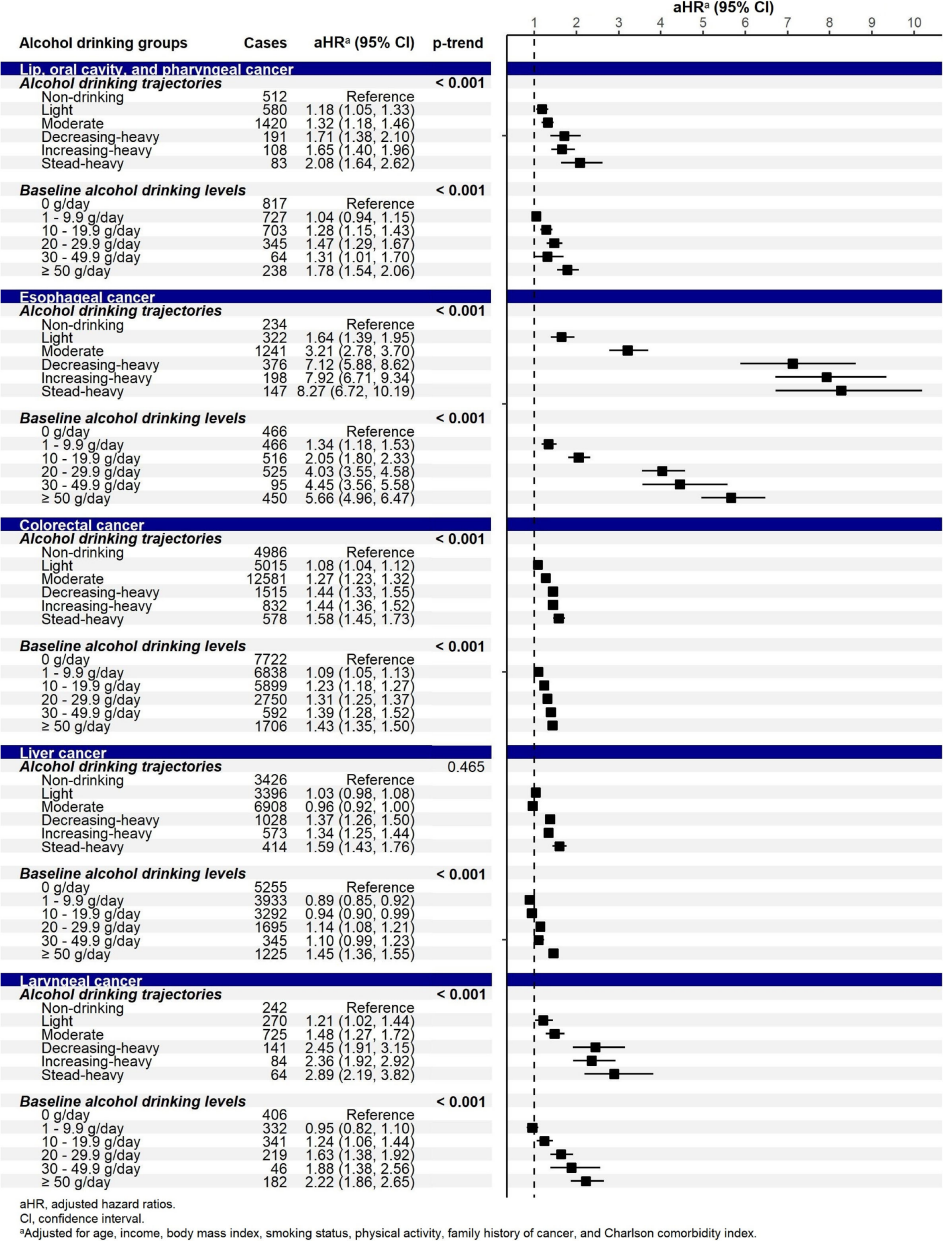
Adjusted hazard ratios (95% confidence interval) for the association between alcohol consumption and the risk for alcohol-related cancers

**Figure 5.**
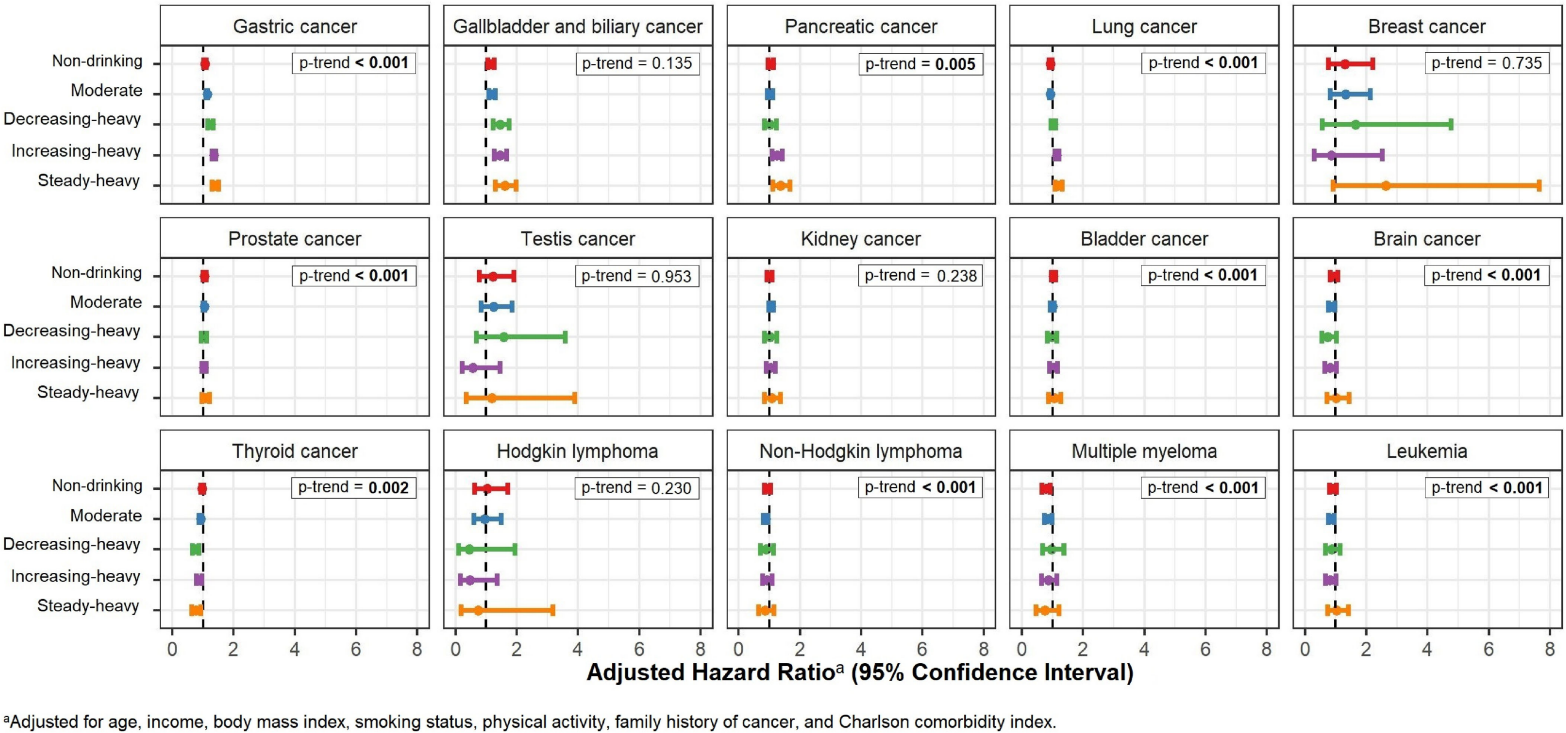
Adjusted hazard ratios (95% confidence interval) for the association between alcohol consumption and the risk for the group of other cancer types

The adjusted hazard ratios (aHRs) with 95% confidence intervals (CIs) for the associations of alcohol consumption with cancer incidence are presented in Figure 3, Figure 4, and Figure 5. For all cancers combined, alcohol intake significantly increased the cancer risk in a dose-dependent manner (ie, the risk increased with the drinking level; Figure 3). Specifically, in the trajectory approach, compared to the TR1 (non-drinking) trajectory, the aHRs were 1.03 (95% CI, 1.02–1.05) for the TR2 (light), 1.06 (95% CI, 1.05–1.08) for the TR3 (moderate), 1.19 (95% CI, 1.16–1.22) for the TR4 (decreasing-heavy), 1.23 (95% CI, 1.20–1.26) for the TR5 (increasing-heavy), and 1.33 (95% CI, 1.29–1.38) for the TR6 (steady-heavy) trajectories (*P*-trend <0.001). In the single-measurement approach, the cancer risk was observed to increase significantly from light alcohol intake (10 g/day) at slightly smaller magnitudes. For the alcohol-related cancer group (Figure 3), the significantly elevated risks were also observed in all trajectories with higher estimates and in a dose-dependent manner (*P*-trend <0.001).

By specific cancers, significant associations were observed in alcohol-related cancers (Figure 4). The estimates of the association were the highest for esophageal cancer, followed by laryngeal cancer. The cancer risk significantly increased for all drinking trajectories for lip, oral cavity, pharyngeal, esophageal, colorectal, and laryngeal cancers. Significant elevation was observed at heavy alcohol intake for liver cancer, specifically in the TR4 (decreasing-heavy), TR5 (increasing-heavy), and TR6 (steady-heavy) trajectories. We also found significant associations for some other cancers whose causal relationships with alcohol consumption have not been established yet (Figure 5, eTable 5). The risk increased by 7–40% for gastric cancer and 15–61% for gallbladder and biliary tract cancers following light intake; it also increased by 25–37% for pancreatic cancer and 15–19% for lung cancer in the TR5 (increasing-heavy) and TR6 (steady-heavy) trajectories. A significant inverse association was observed for thyroid cancer following moderate intake.

Sensitivity analysis revealed that compared to the TR6 (steady-heavy) trajectory, other trajectories were at a significantly lower risk for all cancers and all alcohol-related cancers combined (eTable 6). In particular, the risk for all cancers combined in the TR4 (decreasing-heavy) trajectory significantly decreased by 11–12%. Subgroup analysis of non-smokers supported our main findings (eTable 7). However, for lung cancer, a significant association between alcohol consumption and cancer risk was observed in the TR5 (increasing-heavy) trajectory only. The results after excluding incident cases during 1 year after 2007 were consistent with those of the main analysis (eTable 8). In the analysis of the combined “heavy drinker” group (eTable 9), the pooled risks were significantly increased for pancreatic and lung cancers, which were only found in the TR5 (increasing-heavy) and the TR6 (steady-heavy) trajectories in the main analysis.

## DISCUSSION

We identified significant associations between alcohol consumption trajectories and the risks of all cancers combined, alcohol-related cancers combined (including lip, oral cavity, pharyngeal, esophageal, colorectal, laryngeal, and hepatic cancers),^1^^–^^3^^,^^23^ and several cancers (including stomach, gallbladder, biliary tract, pancreatic, and lung cancers). The previously mentioned Australian study investigating alcohol consumption trajectories over the life-course also observed increased risks of alcohol-related cancers with similar estimates (45–94% increase).^6^ However, the limited number of cancer cases in that study enabled specific evaluations of the association between alcohol consumption and only colorectal cancer.^6^

We noted a significant cancer-risk elevation from light alcohol intake (≥10 g/day), which was previously reported for several cancers (including oropharyngeal, esophageal, and colorectal cancers).^4^^,^^5^^,^^24^^,^^25^ A meta-analysis on lifetime alcohol consumption also supports our findings for upper aero-digestive tract and colorectal cancers.^23^ However, some previous studies on colorectal cancer did not identify a significant relevance to the cancer risk at light alcohol consumption.^26^^,^^27^ Particularly, a meta-analysis of five case-control and 11 nested case-control studies reported a J-shaped association of alcohol consumption with the colorectal cancer risk, in which light-to-moderate drinking (≤28 g/day) was associated with a risk reduction (OR 0.92; 95% CI, 0.88–0.98), compared to non/occasional drinking (≤1 g/day).^26^ The significant associations of low alcohol consumption with laryngeal, gastric, and gallbladder and biliary tract cancers in our study have not been reported previously.^4^^,^^5^^,^^28^ A large sample size might enable us to detect the association at light alcohol intake. The temporal change of alcohol consumption can be captured with multiple measurements in trajectory analyses. For example, among the 891,728 participants consuming 1–9.9 g/day of alcohol at the baseline, 361,122 (40.5%) participants increased their consumption over a 6-year period (data not shown). They were then classified into three trajectories: TR2 (light; 32.7%), TR3 (moderate; 66.2%), and TR4 (increasing-heavy; 1.1%). Therefore, alcohol trajectories provide a useful approach for alcohol consumption classification.

The positive associations of heavy alcohol intake with hepatic and pancreatic cancers noted in this study have also been reported previously.^2^^,^^29^^–^^31^ We found a positive association of heavy drinking with lung cancer, which supports the finding from a meta-analysis that noted a 14% risk increase for >50 g/day of alcohol intake.^2^ Among non-smokers, this association reached statistical significance in only the TR5 (increasing-heavy) trajectory, given the small number of cases in the TR6 (steady-heavy) trajectory (*N* = 73, data not shown). A review on the association between alcohol consumption and lung cancer risk in non-smokers concluded no clear effects of alcohol drinking on cancer risk; however, some of the studies included in the review showed a dose-response relation for total alcohol intake and for spirits.^32^ Further studies that consider the interaction between alcohol consumption and tobacco smoking and adjust for the smoking quantity are required to confirm the findings for this cancer type.

Our findings of the inverse association between alcohol consumption and thyroid cancer risk support the findings of previous studies.^2^ The mechanisms underlying this association remain unclear, although the following biological processes may be involved: reduction in the thyroid-stimulating hormone levels (risk factor for thyroid cancer) and its direct toxic effect on the thyroid cells (leading to thyroid volume reduction).^33^^,^^34^ Previous studies on the thyroid cancer “epidemic” in Korea suggested that the chance of thyroid cancer detection may be higher in never drinkers as they might seek healthcare services more actively.^35^^,^^36^ A positive dose-dependent association between alcohol use and thyroid enlargement was also reported previously.^37^ Thus, the protective effect of alcohol consumption on thyroid cancer should be interpreted cautiously.

Although distinct behavioral patterns identified in the trajectory analyses were specific to sample data,^11^ some of our trajectories were similar to those reported in other studies.^7^^,^^12^ The light, increasing-heavy, and decreasing-heavy trajectories in our study may correspond to the stable low risk, low-to-moderate risk, and moderate-to-low risk classes reported by Baek and Choi (2021),^7^ who analyzed Korean adults over a 11-year-period. Apart from the stable moderate risk class in their study, we further subgrouped moderate and heavy drinkers into two more classes (moderate and steady-heavy drinkers), enabling the detection of risk reduction in decreasing-heavy drinkers, compared with steady-heavy drinkers. In the Australian study, different patterns of heavy drinking were also observed during the life-course.^6^ Alcohol trajectories mapped over a short period, such as in our study, might not reflect the lifetime behavior for an individual. Nevertheless, the trajectory approach offers some key advantages over the single-measurement approach. First, trajectory modelling was performed in consistency with the baseline classification; it was more effective in demonstrating the association of alcohol consumption with the cancer risk at higher magnitudes of estimate. Trajectory analyses over a longer period may potentially reduce the sick-quitter bias caused by heterogeneity of the reference group (lifetime abstainers and former drinkers). Second, trajectory analysis differentiates subgroups of heavy drinkers. Lower risks in decreasing-heavy drinkers as compared to in steady-heavy drinkers implied positive health effects of drinking reduction. Conversely, the trade-off between distinctive model features and group membership should be considered. Provided that moderate and heavy drinkers accounted for only 6% of the population (≥30 g/day) at the baseline, a small group membership in subgroups of heavy drinking was expected; 1% of the study population was included in the model selection criteria instead of 5% applied in other studies.^11^ The sensitivity analysis was performed with the combined group of heavy drinkers, supporting our main findings. Lastly, the model selection criteria seemed subjective, particularly in case of the BICs increasing with the group number.^11^ We evaluated selected models using diagnostic criteria to ensure model quality for further analysis.

Our study has several strengths. Considering that the controversy on safe drinking limits with respect to cancer risks was derived from case-control studies, the population-based cohort design of our study is its strength. A large sample size and a long follow-up period helped examine various cancers. Furthermore, a trajectory analysis was used to capture the temporal alcohol drinking patterns. However, this study also has some limitations. Alcohol consumption was self-reported, so it could be under-reported.^38^ Besides, we were unable to distinguish between former and non-drinkers, because data on the drinking status were not available. Using insurance claims data, we defined cancer cases using ICD-10 codes as those diagnosed primarily and further confirmed by a special code for cancer patients. The reliability of primary cancer diagnosis in the NHIS data was confirmed in a recent study.^39^ Although we adjusted for some important covariates, residual confounders may exist, such as education, diet, and genetics. Future research should investigate drinking trajectories over longer time periods and adjust for confounders more comprehensively for specific cancers.

Alcohol-related health burden, including cancers, is a critical public health concern.^40^ In 2020, alcoholic beverages were globally responsible for 741,300 (4.1%) of new cases of cancers of all types, and the largest burden was present in men (76.7%).^41^ In Korea, men aged ≥15 years consume a yearly average of 16.7 L of pure alcohol (corresponding consumption in the western Pacific region: 7.3 L; 2016).^40^ While alcohol drinking is accepted in the Korean culture, no national action plan has been introduced in the country, making control of alcohol consumption tremendously challenging.^40^ This study provides high-quality evidence in support of alcohol carcinogenicity to humans and policy advocacy. In addition to alcohol-related cancers with established relations, we presented the associations of alcohol drinking with gastric, gallbladder and biliary tract, and pancreatic cancers. Drinking behaviors may change over time, leading to changes in related cancer risks. Therefore, for cancer prevention, initiation of alcohol consumption should be avoided and heavy drinkers should quit as soon as possible.

In conclusion, no safe drinking limits were identified for cancer risk. Light-to-heavy alcohol consumption significantly increased the risk for all cancers combined and for alcohol-related cancers combined in a dose-responsive pattern. Light alcohol consumption was significantly associated with the risk of lip, oral cavity, pharyngeal, esophageal, colorectal, laryngeal, stomach, and gallbladder and biliary tract cancers, and heavy consumption with the risk of hepatic, pancreatic, and lung cancers. Reduction in heavy alcohol consumption showed protective effects.

## SUPPLEMENTARY MATERIAL

eTable 1. BIC estimation in trajectory analysis

eTable 2. Trajectory model selection

eTable 3. Trajectory model evaluation for the selected model (011111)

eTable 4. General characteristics of the study population by baseline alcohol drinking levels

eTable 5. Adjusted hazard ratios^a^ for the association between alcohol consumption and the risk for the group of other cancer types

eTable 6. Sensitivity analysis: Adjusted hazard ratios^a^ for the association between alcohol consumption trajectories and the cancer risk with the steady-heavy trajectory as the reference group

eTable 7. Adjusted hazard ratios^a^ for the association between alcohol consumption and the cancer risk in the subgroup of non-smokers (N = 1,014,304)

eTable 8. Adjusted hazard ratios^a^ for the association between alcohol consumption trajectories and the cancer risk in case of excluding all cancer cases diagnosed within 1 year after the exposure measurement period (2002–2007) (N = 2,821,315)

eTable 9. Adjusted hazard ratios^a^ (95% confidence interval) for the association between alcohol consumption trajectories and the cancer risk with the combination of the three alcohol trajectories into one group
