# Erratum for “Cancer Risk According to Alcohol Consumption Trajectories: A Population-based Cohort Study of 2.8 Million Korean Men”

**DOI:** 10.2188/jea.JE20230158

**Published:** 2023-12-05

**Authors:** Thi Tra Bui, Minji Han, Ngoc Minh Luu, Thi Phuong Thao Tran, Min Kyung Lim, Jin-Kyoung Oh

**Affiliations:** 1 Department of Cancer Control and Population Health, National Cancer Center Graduate School of Cancer Science and Policy, Goyang, Republic of Korea; 2 Division of Cancer Prevention, National Cancer Control Institute, National Cancer Center, Goyang, Republic of Korea; 3 Department of Social and Preventive Medicine, College of Medicine, Inha University, Incheon, Republic of Korea

There were several errors in the original publication of this article.^1^ First, in Table 1 (page 627), the descriptive statistics of the (4) decreasing-heavy and (5) increasing-heavy trajectories on age group, income, body mass index, alcohol drinking, and smoking status were mistakenly transposed. Second, in Figure 4 (page 629), the number of cases in the two trajectories (decreasing-heavy and increasing-heavy) was mistakenly transposed. Third, there were some errors in wording and labeling in the main text. The corrections have been made in the main text as shown in eTable 1, and Table 1 and Figure 4 have been reproduced. The authors apologize for these errors.

**Table 1.** General characteristics of study population at study entry (2002–2003)

	Total (*n* = 2,839,332)	(1) Non-drinking (*n* = 480,832)	(2) Light (*n* = 619,617)	(3) Moderate (*n* = 1,521,465)	(4) Decreasing-heavy (*n* = 60,511)	(5) Increasing-heavy (*n* = 123,122)	(6) Steady-heavy (*n* = 33,785)
**Age group, *N* (%)**							
20–<30 years	414,361 (14.6)	45,013 (9.4)	97,665 (15.8)	250,627 (16.5)	6,482 (10.7)	12,795 (10.4)	1,779 (5.3)
30–<40 years	956,856 (33.7)	123,736 (25.7)	209,055 (33.7)	566,077 (37.2)	15,037 (24.9)	36,342 (29.5)	6,609 (19.6)
40–<50 years	808,558 (28.5)	134,635 (28.0)	167,233 (27.0)	437,288 (28.7)	18,727 (30.9)	39,108 (31.8)	11,567 (34.2)
50–<60 years	403,361 (14.2)	92,814 (19.3)	87,257 (14.1)	182,621 (12.0)	11,397 (18.8)	21,058 (17.1)	8,214 (24.3)
60–<70 years	198,786 (7.0)	61,729 (12.8)	45,033 (7.3)	69,227 (4.6)	6,936 (11.5)	11,251 (9.1)	4,610 (13.6)
≥70 years	57,410 (2.0)	22,905 (4.8)	13,374 (2.2)	15,625 (1.0)	1,932 (3.2)	2,568 (2.1)	1,006 (3.0)

**Income**, ***N* (%)**							
1^st^ quintile	255,292 (9.0)	54,850 (11.4)	57,869 (9.3)	120,829 (7.9)	6,267 (10.4)	12,043 (9.8)	3,434 (10.2)
2^nd^ quintile	390,612 (13.8)	69,942 (14.5)	85,708 (13.8)	201,888 (13.3)	9,667 (16.0)	18,337 (14.9)	5,070 (15.0)
3^rd^ quintile	675,213 (23.8)	109,303 (22.7)	144,799 (23.4)	367,757 (24.2)	14,921 (24.7)	30,311 (24.6)	8,122 (24.0)
4^th^ quintile	700,008 (24.7)	111,855 (23.3)	149,416 (24.1)	385,606 (25.3)	14,283 (23.6)	30,502 (24.8)	8,346 (24.7)
5^th^ quintile	705,478 (24.8)	120,629 (25.1)	155,229 (25.1)	380,311 (25.0)	13,198 (21.8)	28,093 (22.8)	8,018 (23.7)
Missing	112,729 (4.0)	14,253 (3.0)	26,596 (4.3)	65,074 (4.3)	2,175 (3.6)	3,836 (3.1)	795 (2.4)

**Family history of cancer, *N* (%)**	367,548 (12.9)	60,887 (12.7)	73,402 (11.8)	202,716 (13.3)	8,338 (13.8)	17,016 (13.8)	5,189 (15.4)

**Body mass index, *N* (%)**							
<18.5 kg/m^2^	60,652 (2.1)	13,672 (2.8)	14,515 (2.3)	27,146 (1.8)	1,045 (1.7)	1,947 (1.6)	605 (1.8)
18.5–22.9 kg/m^2^	1,023,640 (36.1)	181,328 (37.7)	235,578 (38.0)	536,885 (35.3)	19,235 (31.8)	39,582 (32.1)	11,032 (32.7)
23.0–24.9 kg/m^2^	780,828 (27.5)	128,737 (26.8)	169,391 (27.3)	425,510 (28.0)	15,751 (26.0)	32,711 (26.6)	8,728 (25.8)
≥25 kg/m^2^	972,424 (34.2)	156,248 (32.5)	199,260 (32.2)	530,386 (34.9)	24,402 (40.3)	48,754 (39.6)	13,374 (39.6)
Missing	1,788 (0.1)	847 (0.2)	873 (0.1)	1,538 (0.1)	78 (0.1)	128 (0.1)	46 (0.1)

**Alcohol drinking, *N* (%)**							
0 g/day	810,493 (28.5)	480,832 (100)	247,558 (40.0)	76,925 (5.1)	0 (0)	5,178 (4.2)	0 (0)
1–9.9 g/day	891,728 (31.4)	0 (0)	291,129 (47.0)	590,674 (38.8)	0 (0)	9,925 (8.1)	0 (0)
10–19.9 g/day	738,441 (26.0)	0 (0)	70,383 (11.4)	637,200 (41.9)	0 (0)	30,858 (25.1)	0 (0)
20–29.9 g/day	227,533 (8.0)	0 (0)	8,608 (1.4)	175,108 (11.5)	0 (0)	43,817 (35.6)	0 (0)
30–49.9 g/day	63,085 (2.2)	0 (0)	1,939 (0.3)	32,436 (2.1)	7,436 (12.3)	21,274 (17.3)	0 (0)
≥50 g/day	108,052 (3.8)	0 (0)	0 (0)	9,122 (0.6)	53,075 (87.7)	12,070 (9.8)	33,785 (100)

**Smoking status,** *n* (%)							
Never smoker	1,014,304 (35.7)	269,547 (56.1)	270,465 (43.7)	427,420 (28.1)	12,790 (21.1)	27,151 (22.1)	6,931 (20.5)
Former smoker	445,732 (15.7)	56,795 (11.8)	94,035 (15.2)	261,963 (17.2)	8,867 (14.7)	18,989 (15.4)	5,083 (15.0)
Current smoker	1,367,240 (48.2)	152,571 (31.7)	251,396 (40.6)	826,422 (54.3)	38,684 (63.9)	76,471 (62.1)	21,696 (64.2)
Missing	12,056 (0.4)	1,919 (0.4)	3,721 (0.6)	5,660 (0.4)	170 (0.3)	511 (0.4)	75 (0.2)

**Physical exercise,** *n* (%)							
0 times/week	1,238,772 (43.6)	232,243 (48.3)	280,387 (45.3)	616,044 (40.5)	31,266 (51.7)	59,968 (48.7)	18,864 (55.8)
1–2 times/week	997,719 (35.1)	145,246 (30.2)	210,266 (33.9)	581,584 (38.2)	15,828 (26.2)	37,077 (30.1)	7,718 (22.8)
3–4 times/week	326,480 (11.5)	52,016 (10.8)	69,234 (11.2)	182,818 (12)	6,027 (10)	13,311 (10.8)	3,074 (9.1)
5–6 times/week	71,344 (2.5)	12,185 (2.5)	14,823 (2.4)	38,482 (2.5)	1,592 (2.6)	3,325 (2.7)	937 (2.8)
Almost everyday	132,090 (4.7)	28,570 (5.9)	27,951 (4.5)	61,839 (4.1)	4,540 (7.5)	6,572 (5.3)	2,618 (7.7)
Missing	72,927 (2.6)	10,572 (2.2)	16,956 (2.7)	40,698 (2.7)	1,258 (2.1)	2,869 (2.3)	574 (1.7)

**Charlson Comorbidity Index,** *n* (%)							
0	2,805,892 (98.8)	471,826 (98.1)	611,025 (98.6)	1,508,451 (99.1)	59,639 (98.6)	121,619 (98.8)	33,332 (98.7)
1	27,490 (1.0)	7,055 (1.5)	6,990 (1.1)	11,120 (0.7)	717 (1.2)	1,247 (1.0)	361 (1.1)
2	4,358 (0.2)	1,337 (0.3)	1,177 (0.2)	1,458 (0.1)	118 (0.2)	195 (0.2)	73 (0.2)
≥3	1,592 (0.1)	614 (0.1)	425 (0.1)	436 (0.1)	37 (0.1)	61 (0)	19 (0.1)

**Figure 4.**
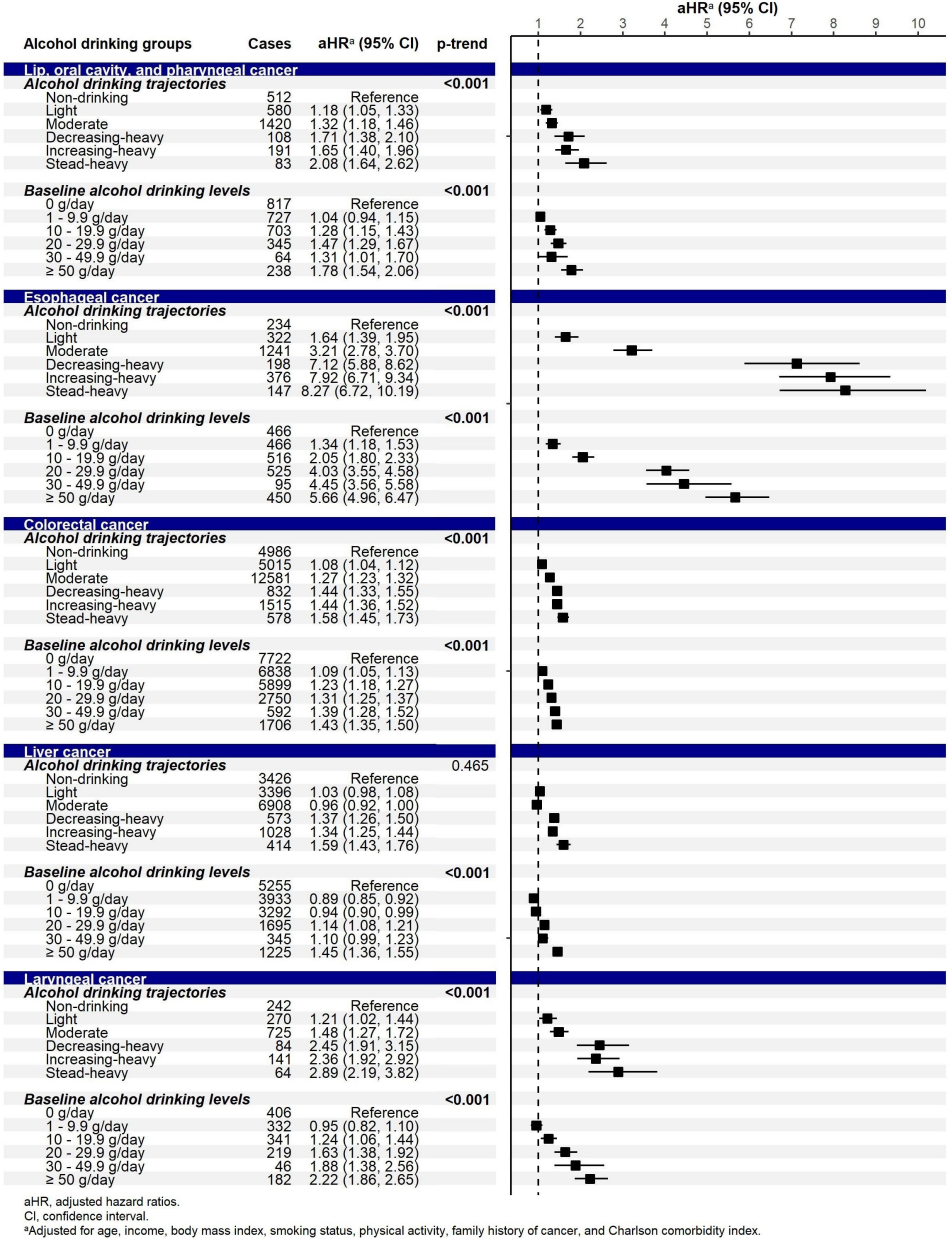
Adjusted hazard ratios (95% confidence interval) for the association between alcohol consumption and the risk for alcohol-related cancers

## SUPPLEMENTARY MATERIAL

eTable 1. Erratum in the main text
