# Supplementary material for: Cancer Risk According to Alcohol Consumption Trajectories: A Population-based Cohort Study of 2.8 Million Korean Men
Source: J Epidemiol. 2023 Dec 5;33(12):624–32. doi: 10.2188/jea.JE20220175 (PMC10635810; doi:10.2188/jea.JE20220175)
Supplement: Supplementary file 1 [file je-33-624-s001.pdf]

eTable 1. BIC estimation in trajectory analysis

**Number of groups**	**BIC** (n=8,517,996) (order=2)	**BIC** (n=2,839,332) (order=2)	**Selected** (Yes/No)	**Best fit model**	**BIC** (n=8,517,996)	**BIC** (n=2,839,332)
**1**	-14077697	-14077695	No			
**2**	-13102690	-13102685	No			
**3**	-12634690	-12634683	No			
**4**	-12557221	-12557212	No			
**5**	-12467490	-12467479	No			
**6**	-12408592	-12408578	Yes	0 1 1 1 1 1	-12417347	-12417338
**7**	-12343456	-12343440	No			

BIC, Bayesian information criterion.

eTable 2. Trajectory model selection

Number of groups	Selected (Yes/No)	Selection criteria for the group number			
		BIC	Parsimony	Group membership ≥1% in all groups	Distinctive features
**1**	No		+	+	
**2**	No		+	+	
**3**	Yes		+	+	
**4**	No	+		+	+
**5**	No	+		+	+
**6**	Yes	+		+	++
**7**	No	+		+	++

BIC, Bayesian information criterion.

eTable 3. Trajectory model evaluation for the selected model (011111)

Alcohol drinking trajectories	N (%)	Estimated group membership	Average posterior probability of assignment	Odds of correct classification
**Non-drinking**	480,832 (16.9%)	16.1%	0.95	95.3
**Light**	619,617 (21.8%)	28.8%	0.83	12.3
**Moderate**	1,521,465 (53.6%)	47.2%	0.80	4.5
**Decreasing-heavy**	60,511 (2.1%)	2.2%	0.85	119.8
**Increasing-heavy**	123,122 (4.4%)	4.5%	0.86	261.1
**Steady-heavy**	33,785 (1.2%)	1.2%	0.90	736.8

eTable 4. General characteristics of the study population by baseline alcohol drinking levels

	(1)<br>0 g/day	(2)<br>1–9.9 g/day	(3)<br>10–19.9 g/day	(4)<br>20–29.9 g/day	(5)<br>30–49.9 g/day	(6)<br>≥50 g/day
**N (%)**	810,493 (28.5)	891,728 (31.4)	738,441 (26.0)	227,533 (8.0)	63,085 (2.2)	108,052 (3.8)
**Age group, N (%)**						
**20 - <30 years**	96,339 (11.9)	150,473 (16.9)	125,002 (16.9)	25,182 (11.1)	8,783 (13.9)	8,582 (7.9)
**30 - <40 years**	235,365 (29.0)	331,504 (37.2)	279,636 (37.9)	65,541 (28.8)	21,914 (34.7)	22,896 (21.2)
**40 - <50 years**	223,741 (27.6)	240,439 (27.0)	217,135 (29.4)	71,272 (31.3)	22,110 (35.0)	33,861 (31.3)
**50 - <60 years**	138,545 (17.1)	110,678 (12.4)	83,762 (11.3)	38,606 (17.0)	8,047 (12.8)	23,723 (22.0)
**60 - <70 years**	86,389 (10.7)	46,930 (5.3)	27,626 (3.7)	20,800 (9.1)	2,009 (3.2)	15,032 (13.9)
**≥70 years**	30,114 (3.7)	11,704 (1.3)	5,280 (0.7)	6,132 (2.7)	222 (0.4)	3,958 (3.7)
**Income, N (%)**						
**1st quintile**	86,799 (10.7)	72,132 (8.1)	57,629 (7.8)	22,051 (9.7)	5,140 (8.1)	11,541 (10.7)
**2nd quintile**	117,168 (14.5)	115,937 (13.0)	99,247 (13.4)	32,498 (14.3)	8,522 (13.5)	17,240 (16.0)
**3rd quintile**	187,051 (23.1)	211,039 (23.7)	181,093 (24.5)	54,592 (24.0)	15,068 (23.9)	26,370 (24.4)
**4th quintile**	191,560 (23.6)	223,645 (25.1)	187,157 (25.3)	55,786 (24.5)	16,181 (25.6)	25,679 (23.8)
**5th quintile**	201,647 (24.9)	229,640 (25.8)	180,187 (24.4)	54,392 (23.9)	15,741 (25.0)	23,871 (22.1)
**Missing**	26,268 (3.2)	39,335 (4.4)	33,128 (4.5)	8,214 (3.6)	2,433 (3.9)	3,351 (3.1)
**Family history of cancer, N (%)**	94,901 (10.6)	113,642 (12.7)	101,790 (13.8)	32,330 (14.2)	9,814 (15.6)	15,071 (13.9)
**Body mass index, N (%)**						
**<18.5 kg/m²**	21,665 (2.7)	19,025 (2.1)	11,642 (1.6)	3,866 (1.7)	729 (1.2)	2,003 (1.9)
**18.5–22.9 kg/m²**	306,275 (37.8)	340,119 (38.1)	248,857 (33.7)	74,749 (32.9)	18,094 (28.7)	35,546 (32.9)
**22.9–24.9 kg/m²**	217,931 (26.9)	249,524 (28.0)	206,944 (28.0)	61,431 (27.0)	17,083 (27.1)	27,915 (25.8)
**≥25 kg/m²**	263,268 (32.5)	282,051 (31.6)	270,301 (36.6)	87,246 (38.3)	27,121 (43.0)	42,437 (39.3)
**Missing**	1,354 (0.1)	1,009 (0.1)	697 (0.1)	241 (0.1)	58 (0.1)	151 (0.1)
**Alcohol drinking, N (%)**						
**0 g/day**	810,493 (100)	0 (0)	0 (0)	0 (0)	0 (0)	0 (0)
**1–9.9 g/day**	0 (0)	891,728 (100)	0 (0)	0 (0)	0 (0)	0 (0)
**10–19.9 g/day**	0 (0)	0 (0)	738,441 (100)	0 (0)	0 (0)	0 (0)

	(1)<br>0 g/day	(2)<br>1–9.9 g/day	(3)<br>10–19.9 g/day	(4)<br>20–29.9 g/day	(5)<br>30–49.9 g/day	(6)<br>≥50 g/day
**20–29.9 g/day**	0 (0)	0 (0)	0 (0)	227,533 (100)	0 (0)	0 (0)
**30–49.9 g/day**	0 (0)	0 (0)	0 (0)	0 (0)	63,085 (100)	0 (0)
**≥50 g/day**	0 (0)	0 (0)	0 (0)	0 (0)	0 (0)	108,052 (100)
**Smoking status, N (%)**						
**Never smoker**	466,255 (57.5)	292,039 (32.7)	171,479 (23.2)	49,930 (21.9)	11,136 (17.7)	23,465 (21.7)
**Former smoker**	95,889 (11.8)	160,599 (18.0)	125,298 (17.0)	37,639 (16.5)	10,290 (16.3)	16,017 (14.8)
**Current smoker**	243,215 (30.0)	435,191 (48.8)	439,780 (59.6)	139,335 (61.2)	41,469 (65.7)	68,250 (63.2)
**Missing**	5,134 (0.6)	3,899 (0.5)	1,884 (0.2)	629 (0.4)	190 (0.3)	320 (0.3)
**Physical exercise, N (%)**						
**0 times/week**	407,111 (45.7)	363,062 (40.7)	281,443 (38.1)	102,233 (44.9)	25,745 (40.8)	59,178 (54.8)
**1–2 times/week**	237,116 (26.6)	344,057 (38.6)	295,407 (40.0)	73,026 (32.1)	22,677 (35.9)	25,436 (23.5)
**3–4 times/week**	82,610 (9.3)	105,445 (11.8)	93,041 (12.6)	26,924 (11.8)	8,601 (13.6)	9,859 (9.1)
**5–6 times/week**	18,618 (2.1)	22,763 (2.6)	18,832 (2.6)	6,440 (2.8)	1,902 (3.0)	2,789 (2.6)
**Almost every day**	41,991 (4.7)	36,322 (4.1)	28,998 (3.9)	13,474 (5.9)	2,484 (3.9)	8,821 (8.2)
**Missing**	23,047 (2.6)	20,079 (2.3)	20,720 (2.8)	5,436 (2.4)	1,676 (2.7)	1,969 (1.8)
**Charlson Comorbidity Index, N (%)**						
**0**	796,684 (98.3)	883,455 (99.1)	732,085 (99.1)	224,906 (98.8)	62,464 (99.0)	106,298 (98.4)
**1**	10,959 (1.4)	6,981 (0.8)	5,446 (0.7)	2,168 (1.0)	525 (0.8)	1411 (1.3)
**2**	2,005 (0.2)	997 (0.1)	695 (0.1)	327 (0.1)	70 (0.1)	264 (0.2)
**≥3**	845 (0.1)	295 (0)	215 (0.1)	132 (0.1)	26 (0.1)	79 (0.1)

eTable 5. Adjusted hazard ratios[a] for the association between alcohol consumption and the risk for the group of other cancer types

Cancer types	Alcohol drinking trajectories		Baseline alcohol drinking levels	
	Groups	aHR (95% CI)	Groups	aHR (95% CI)
**Gastric cancer**	Non-drinking	Reference	0 g/day	Reference
	Light	1.07 (1.03–1.10)	1–9.9 g/day	1.00 (0.98–1.03)
	Moderate	1.14 (1.11–1.17)	10–19.9 g/day	1.10 (1.07–1.13)
	Decreasing-heavy	1.25 (1.17–1.33)	20–29.9 g/day	1.17 (1.12–1.21)
	Increasing-heavy	1.35 (1.29–1.42)	30–49.9 g/day	1.17 (1.08–1.25)
	Steady-heavy	1.40 (1.30–1.51)	≥50 g/day	1.26 (1.21–1.32)
**Gallbladder and biliary tract cancer**	Non-drinking	Reference	0 g/day	Reference
	Light	1.15 (1.05–1.26)	1–9.9 g/day	1.07 (0.98–1.16)
	Moderate	1.20 (1.10–1.30)	10–19.9 g/day	1.08 (0.99–1.19)
	Decreasing-heavy	1.46 (1.23–1.75)	20–29.9 g/day	1.35 (1.22–1.50)
	Increasing-heavy	1.46 (1.27–1.68)	30–49.9 g/day	1.16 (0.91–1.48)
	Steady-heavy	1.61 (1.31–1.98)	≥50 g/day	1.58 (1.40–1.79)
**Pancreatic cancer**	Non-drinking	Reference	0 g/day	Reference
	Light	1.05 (0.96–1.14)	1–9.9 g/day	0.97 (0.90–1.05)
	Moderate	1.03 (0.95–1.12)	10–19.9 g/day	1.03 (0.94–1.12)
	Decreasing-heavy	1.03 (0.85–1.24)	20–29.9 g/day	1.04 (0.93–1.16)
	Increasing-heavy	1.25 (1.09–1.43)	30–49.9 g/day	1.18 (0.96–1.46)
	Steady-heavy	1.37 (1.12–1.67)	≥50 g/day	1.09 (0.96–1.24)
**Lung cancer**	Non-drinking	Reference	0 g/day	Reference
	Light	0.94 (0.90–0.98)	1–9.9 g/day	0.85 (0.82–0.88)
	Moderate	0.94 (0.91–0.97)	10–19.9 g/day	0.91 (0.87–0.95)
	Decreasing-heavy	1.03 (0.95–1.11)	20–29.9 g/day	0.98 (0.94–1.03)
	Increasing-heavy	1.15 (1.08–1.22)	30–49.9 g/day	1.10 (0.99–1.21)
	Steady-heavy	1.19 (1.09–1.31)	≥50 g/day	1.13 (1.07–1.20)
**Breast cancer**	Non-drinking	Reference	0 g/day	Reference
	Light	1.30 (0.76–2.21)	1–9.9 g/day	1.08 (0.68–1.71)
	Moderate	1.33 (0.82–2.14)	10–19.9 g/day	1.04 (0.62–1.74)
	Decreasing-heavy	1.65 (0.57–4.77)	20–29.9 g/day	1.60 (0.87–2.92)
	Increasing-heavy	0.87 (0.30–2.53)	30–49.9 g/day	1.48 (0.45–4.85)
	Steady-heavy	2.64 (0.91–7.66)	≥50 g/day	2.62 (1.38–4.96)
**Prostate cancer**	Non-drinking	Reference	0 g/day	Reference
	Light	1.05 (1.01–1.10)	1–9.9 g/day	1.02 (0.98–1.06)
	Moderate	1.04 (1.00–1.07)	10–19.9 g/day	1.04 (1.00–1.09)
	Decreasing-heavy	1.03 (0.94–1.13)	20–29.9 g/day	1.01 (0.96–1.07)
	Increasing-heavy	1.03 (0.96–1.10)	30–49.9 g/day	1.05 (0.93–1.18)
	Steady-heavy	1.08 (0.97–1.21)	≥50 g/day	1.02 (0.96–1.09)

**Testis cancer**	Non-drinking	Reference	0 g/day	Reference
	Light	1.23 (0.79–1.90)	1–9.9 g/day	1.67 (1.17–2.38)
	Moderate	1.25 (0.84–1.84)	10–19.9 g/day	1.19 (0.80–1.79)
	Decreasing-heavy	1.58 (0.69–3.58)	20–29.9 g/day	1.66 (1.01–2.74)
	Increasing-heavy	0.57 (0.22–1.45)	30–49.9 g/day	0.77 (0.24–2.49)
	Steady-heavy	1.19 (0.36–3.89)	≥50 g/day	1.42 (0.71–2.82)
**Kidney cancer**	Non-drinking	Reference	0 g/day	Reference
	Light	1.00 (0.92–1.10)	1–9.9 g/day	1.02 (0.94–1.10)
	Moderate	1.05 (0.98–1.14)	10–19.9 g/day	1.08 (1.00–1.17)
	Decreasing-heavy	1.03 (0.85–1.25)	20–29.9 g/day	1.05 (0.94–1.17)
	Increasing-heavy	1.04 (0.90–1.20)	30–49.9 g/day	1.06 (0.88–1.29)
	Steady-heavy	1.08 (0.85–1.37)	≥50 g/day	1.00 (0.87–1.16)
**Bladder cancer**	Non-drinking	Reference	0 g/day	Reference
	Light	1.03 (0.96–1.11)	1–9.9 g/day	0.93 (0.87–0.99)
	Moderate	1.00 (0.94–1.07)	10–19.9 g/day	0.97 (0.90–1.05)
	Decreasing-heavy	0.98 (0.83–1.15)	20–29.9 g/day	0.99 (0.91–1.09)
	Increasing-heavy	1.03 (0.91–1.16)	30–49.9 g/day	0.83 (0.68–1.03)
	Steady-heavy	1.06 (0.88–1.28)	≥50 g/day	0.97 (0.87–1.09)
**Brain cancer**	Non-drinking	Reference	0 g/day	Reference
	Light	0.95 (0.83–1.08)	1–9.9 g/day	0.87 (0.77–0.97)
	Moderate	0.86 (0.76–0.97)	10–19.9 g/day	0.96 (0.85–1.09)
	Decreasing-heavy	0.75 (0.55–1.04)	20–29.9 g/day	0.83 (0.70–0.99)
	Increasing-heavy	0.82 (0.65–1.04)	30–49.9 g/day	1.15 (0.86–1.53)
	Steady-heavy	1.02 (0.72–1.44)	≥50 g/day	0.83 (0.66–1.04)
**Thyroid cancer**	Non-drinking	Reference	0 g/day	Reference
	Light	0.97 (0.92–1.02)	1–9.9 g/day	1.01 (0.96–1.05)
	Moderate	0.93 (0.88–0.97)	10–19.9 g/day	0.91 (0.87–0.95)
	Decreasing-heavy	0.76 (0.66–0.87)	20–29.9 g/day	0.91 (0.85–0.97)
	Increasing-heavy	0.88 (0.80–0.97)	30–49.9 g/day	0.97 (0.87–1.08)
	Steady-heavy	0.77 (0.64–0.92)	≥50 g/day	0.80 (0.72–0.89)
**Hodgkin lymphoma**	Non-drinking	Reference	0 g/day	Reference
	Light	1.03 (0.62–1.71)	1–9.9 g/day	1.12 (0.74–1.68)
	Moderate	0.95 (0.61–1.49)	10–19.9 g/day	0.76 (0.48–1.23)
	Decreasing-heavy	0.46 (0.11–1.95)	20–29.9 g/day	1.02 (0.57–1.83)
	Increasing-heavy	0.48 (0.17–1.37)	30–49.9 g/day	0.25 (0.03–1.85)
	Steady-heavy	0.75 (0.18–3.18)	≥50 g/day	0.49 (0.17–1.37)
**Non-Hodgkin lymphoma**	Non-drinking	Reference	0 g/day	Reference
	Light	0.94 (0.85–1.04)	1–9.9 g/day	0.95 (0.87–1.03)
	Moderate	0.89 (0.81–0.97)	10–19.9 g/day	0.86 (0.78–0.95)
	Decreasing-heavy	0.91 (0.72–1.14)	20–29.9 g/day	0.89 (0.78–1.01)
	Increasing-heavy	0.93 (0.78–1.10)	30–49.9 g/day	1.01 (0.80–1.29)

	Steady-heavy	0.87 (0.65–1.16)	≥50 g/day	0.91 (0.77–1.08)
**Multiple myeloma and malignant plasma cell neoplasms**	Non-drinking	Reference	0 g/day	Reference
	Light	0.79 (0.66–0.93)	1–9.9 g/day	0.90 (0.78–1.04)
	Moderate	0.85 (0.74–0.99)	10–19.9 g/day	0.85 (0.72–1.01)
	Decreasing-heavy	0.96 (0.67–1.37)	20–29.9 g/day	0.80 (0.64–1.00)
	Increasing-heavy	0.87 (0.65–1.15)	30–49.9 g/day	0.55 (0.31–0.95)
	Steady-heavy	0.76 (0.46–1.23)	≥50 g/day	0.91 (0.70–1.19)
**Leukemia**	Non-drinking	Reference	0 g/day	Reference
	Light	0.92 (0.81–1.03)	1–9.9 g/day	0.93 (0.83–1.03)
	Moderate	0.85 (0.77–0.95)	10–19.9 g/day	0.80 (0.71–0.90)
	Decreasing-heavy	0.88 (0.67–1.15)	20–29.9 g/day	0.92 (0.79–1.08)
	Increasing-heavy	0.82 (0.66–1.01)	30–49.9 g/day	0.67 (0.48–0.93)
	Steady-heavy	1.03 (0.75–1.42)	≥50 g/day	1.00 (0.82–1.21)

aHR, adjusted hazard ratios; CI, confidence interval.

[a]Adjusted for age, income, body mass index, smoking status, physical activity, family history of cancer, and Charlson comorbidity index.

eTable 6. Sensitivity analysis: Adjusted hazard ratios[a] for the association between alcohol consumption trajectories and the cancer risk with the steady-heavy trajectory as the reference group

Cancer types	Alcohol drinking trajectories	aHR (95% CI)
**All cancers combined**	Non-drinking	0.75 (0.73–0.78)
	Light	0.78 (0.75–0.80)
	Moderate	0.80 (0.77–0.83)
	Decreasing-heavy	0.89 (0.86–0.93)
	Increasing-heavy	0.92 (0.89–0.96)
	Steady-heavy	Reference
**Alcohol-related cancers combined**	Non-drinking	0.55 (0.52–0.58)
	Light	0.60 (0.56–0.63)
	Moderate	0.67 (0.63–0.70)
	Decreasing-heavy	0.88 (0.82–0.94)
	Increasing-heavy	0.88 (0.82–0.94)
	Steady-heavy	Reference
**Lip, oral cavity, and pharyngeal cancer**	Non-drinking	0.48 (0.38–0.61)
	Light	0.57 (0.45–0.72)
	Moderate	0.63 (0.51–0.79)
	Decreasing-heavy	0.82 (0.62–1.09)
	Increasing-heavy	0.80 (0.62–1.03)
	Steady-heavy	Reference
**Esophageal cancer**	Non-drinking	0.12 (0.10–0.15)
	Light	0.20 (0.16–0.24)
	Moderate	0.39 (0.33–0.46)
	Decreasing-heavy	0.86 (0.70–1.07)
	Increasing-heavy	0.96 (0.79–1.16)
	Steady-heavy	Reference
**Gastric cancer**	Non-drinking	0.71 (0.66–0.77)
	Light	0.76 (0.71–0.82)
	Moderate	0.81 (0.76–0.87)
	Decreasing-heavy	0.89 (0.81–0.98)
	Increasing-heavy	0.97 (0.89–1.05)
	Steady-heavy	Reference
**Colorectal cancer**	Non-drinking	0.63 (0.58–0.69)
	Light	0.68 (0.62–0.74)
	Moderate	0.80 (0.74–0.87)
	Decreasing-heavy	0.91 (0.82–1.01)
	Increasing-heavy	0.91 (0.82–1.00)

	Steady-heavy	Reference
**Liver cancer**	Non-drinking	0.63 (0.57–0.70)
	Light	0.65 (0.59–0.72)
	Moderate	0.61 (0.55–0.67)
	Decreasing-heavy	0.87 (0.76–0.98)
	Increasing-heavy	0.85 (0.76–0.95)
	Steady-heavy	Reference
**Gallbladder and biliary tract cancer**	Non-drinking	0.62 (0.50–0.76)
	Light	0.71 (0.58–0.87)
	Moderate	0.74 (0.61–0.91)
	Decreasing-heavy	0.91 (0.70–1.17)
	Increasing-heavy	0.91 (0.72–1.14)
	Steady-heavy	Reference
**Pancreatic cancer**	Non-drinking	0.73 (0.60–0.89)
	Light	0.76 (0.62–0.93)
	Moderate	0.75 (0.62–0.92)
	Decreasing-heavy	0.75 (0.58–0.97)
	Increasing-heavy	0.91 (0.73–1.14)
	Steady-heavy	Reference
**Laryngeal cancer**	Non-drinking	0.35 (0.26–0.46)
	Light	0.42 (0.32–0.55)
	Moderate	0.51 (0.39–0.66)
	Decreasing-heavy	0.85 (0.61–1.17)
	Increasing-heavy	0.82 (0.61–1.10)
	Steady-heavy	Reference
**Lung cancer**	Non-drinking	0.84 (0.77–0.92)
	Light	0.79 (0.72–0.86)
	Moderate	0.79 (0.72–0.86)
	Decreasing-heavy	0.86 (0.77–0.96)
	Increasing-heavy	0.96 (0.87–1.06)
	Steady-heavy	Reference
**Breast cancer**	Non-drinking	0.38 (0.13–1.10)
	Light	0.49 (0.17–1.40)
	Moderate	0.50 (0.18–1.39)
	Decreasing-heavy	0.62 (0.16–2.49)
	Increasing-heavy	0.33 (0.08–1.32)
	Steady-heavy	Reference
**Prostate cancer**	Non-drinking	0.92 (0.83–1.03)
	Light	0.97 (0.87–1.09)
	Moderate	0.96 (0.86–1.07)

	Decreasing-heavy	0.95 (0.83–1.09)
	Increasing-heavy	0.95 (0.84–1.08)
	Steady-heavy	Reference
**Testis cancer**	Non-drinking	0.84 (0.26–2.75)
	Light	1.03 (0.32–3.32)
	Moderate	1.05 (0.33–3.31)
	Decreasing-heavy	1.33 (0.34–5.13)
	Increasing-heavy	0.47 (0.11–1.99)
	Steady-heavy	Reference
**Kidney cancer**	Non-drinking	0.93 (0.73–1.17)
	Light	0.93 (0.74–1.17)
	Moderate	0.98 (0.78–1.23)
	Decreasing-heavy	0.96 (0.72–1.28)
	Increasing-heavy	0.96 (0.74–1.24)
	Steady-heavy	Reference
**Bladder cancer**	Non-drinking	0.94 (0.78–1.13)
	Light	0.97 (0.80–1.17)
	Moderate	0.94 (0.78–1.13)
	Decreasing-heavy	0.92 (0.73–1.16)
	Increasing-heavy	0.96 (0.78–1.19)
	Steady-heavy	Reference
**Brain cancer**	Non-drinking	0.98 (0.69–1.40)
	Light	0.93 (0.66–1.32)
	Moderate	0.85 (0.60–1.19)
	Decreasing-heavy	0.74 (0.47–1.17)
	Increasing-heavy	0.81 (0.54–1.20)
	Steady-heavy	Reference
**Thyroid cancer**	Non-drinking	1.30 (1.08–1.56)
	Light	1.26 (1.05–1.51)
	Moderate	1.20 (1.01–1.44)
	Decreasing-heavy	0.99 (0.79–1.23)
	Increasing-heavy	1.15 (0.94–1.39)
	Steady-heavy	Reference
**Hodgkin lymphoma**	Non-drinking	1.32 (0.31–5.59)
	Light	1.37 (0.33–5.71)
	Moderate	1.26 (0.31–5.15)
	Decreasing-heavy	0.61 (0.09–4.34)
	Increasing-heavy	0.63 (0.12–3.46)
	Steady-heavy	Reference
	Non-drinking	1.15 (0.86–1.54)

**Non-Hodgkin lymphoma**	Light	1.08 (0.81–1.45)
	Moderate	1.03 (0.77–1.37)
	Decreasing-heavy	1.04 (0.73–1.49)
	Increasing-heavy	1.07 (0.77–1.48)
	Steady-heavy	Reference
**Multiple myeloma and malignant plasma cell neoplasms**	Non-drinking	1.32 (0.81–2.16)
	Light	1.04 (0.64–1.71)
	Moderate	1.13 (0.70–1.83)
	Decreasing-heavy	1.27 (0.71–2.27)
	Increasing-heavy	1.15 (0.67–1.97)
	Steady-heavy	Reference
**Leukemia**	Non-drinking	0.97 (0.71–1.33)
	Light	0.89 (0.65–1.22)
	Moderate	0.83 (0.61–1.13)
	Decreasing-heavy	0.85 (0.57–1.26)
	Increasing-heavy	0.79 (0.55–1.13)
	Steady-heavy	Reference

aHR, adjusted hazard ratios; CI, confidence interval.
[a]Adjusted for age, income, body mass index, smoking status, physical activity, family history of cancer, and Charlson comorbidity index.

eTable 7. Adjusted hazard ratios[a] for the association between alcohol consumption and the cancer risk in the subgroup of non-smokers (N=1,014,304)

Cancer types	Alcohol drinking trajectories		Baseline alcohol drinking levels	
	**Groups**	**aHR (95% CI)**	**Groups**	**aHR (95% CI)**
**All cancers combined**	Non-drinking	Reference	0 g/day	Reference
	Light	1.02 (1.00–1.04)	1–9.9 g/day	0.97 (0.96–0.99)
	Moderate	1.06 (1.04–1.08)	10–19.9 g/day	1.04 (1.02–1.06)
	Decreasing-heavy	1.15 (1.09–1.21)	20–29.9 g/day	1.09 (1.05–1.12)
	Increasing-heavy	1.24 (1.20–1.30)	30–49.9 g/day	1.11 (1.03–1.19)
	Steady-heavy	1.27 (1.18–1.36)	≥50 g/day	1.17 (1.12–1.22)
**Alcohol-related cancers combined**	Non-drinking	Reference	0 g/day	Reference
	Light	1.02 (0.98–1.06)	1–9.9 g/day	0.99 (0.95–1.02)
	Moderate	1.15 (1.11–1.20)	10–19.9 g/day	1.12 (1.07–1.17)
	Decreasing-heavy	1.56 (1.42–1.72)	20–29.9 g/day	1.26 (1.19–1.33)
	Increasing-heavy	1.55 (1.44–1.67)	30–49.9 g/day	1.32 (1.15–1.51)
	Steady-heavy	1.66 (1.47–1.87)	≥50 g/day	1.52 (1.42–1.63)
**Lip, oral cavity, and pharyngeal cancer**	Non-drinking	Reference	0 g/day	Reference
	Light	1.10 (0.92–1.30)	1–9.9 g/day	1.01 (0.87–1.19)
	Moderate	1.23 (1.05–1.44)	10–19.9 g/day	1.16 (0.97–1.40)
	Decreasing-heavy	1.41 (0.91–2.18)	20–29.9 g/day	1.41 (1.11–1.77)
	Increasing-heavy	1.69 (1.25–2.28)	30–49.9 g/day	0.85 (0.42–1.71)
	Steady-heavy	1.63 (0.97–2.75)	≥50 g/day	1.49 (1.11–2.00)
**Esophageal cancer**	Non-drinking	Reference	0 g/day	Reference
	Light	1.75 (1.37–2.24)	1–9.9 g/day	1.53 (1.26–1.86)
	Moderate	3.30 (2.67–4.08)	10–19.9 g/day	1.85 (1.47–2.32)
	Decreasing-heavy	7.38 (5.25–10.36)	20–29.9 g/day	3.19 (2.54–4.00)
	Increasing-heavy	7.54 (5.69–9.99)	30–49.9 g/day	4.47 (2.77–7.23)
	Steady-heavy	6.22 (3.98–9.74)	≥50 g/day	4.81 (3.78–6.11)
**Gastric cancer**	Non-drinking	Reference	0 g/day	Reference
	Light	1.07 (1.02–1.12)	1–9.9 g/day	0.97 (0.93–1.01)
	Moderate	1.13 (1.08–1.18)	10–19.9 g/day	1.09 (1.04–1.15)
	Decreasing-heavy	1.13 (0.99–1.29)	20–29.9 g/day	1.14 (1.06–1.22)
	Increasing-heavy	1.42 (1.30–1.55)	30–49.9 g/day	1.06 (0.89–1.25)
	Steady-heavy	1.45 (1.25–1.69)	≥50 g/day	1.24 (1.14–1.36)
**Colorectal cancer**	Non-drinking	Reference	0 g/day	Reference
	Light	1.03 (0.98–1.09)	1–9.9 g/day	1.05 (1.00–1.10)
	Moderate	1.26 (1.20–1.32)	10–19.9 g/day	1.25 (1.18–1.33)
	Decreasing-heavy	1.52 (1.33–1.74)	20–29.9 g/day	1.26 (1.17–1.36)

	Increasing-heavy	1.45 (1.31–1.60)	30–49.9 g/day	1.45 (1.22–1.72)
	Steady-heavy	1.50 (1.26–1.78)	≥50 g/day	1.40 (1.27–1.55)
**Liver cancer**	Non-drinking	Reference	0 g/day	Reference
	Light	0.94 (0.87–1.00)	1–9.9 g/day	0.85 (0.79–0.91)
	Moderate	0.87 (0.81–0.92)	10–19.9 g/day	0.86 (0.79–0.93)
	Decreasing-heavy	1.28 (1.08–1.53)	20–29.9 g/day	1.05 (0.94–1.16)
	Increasing-heavy	1.34 (1.18–1.52)	30–49.9 g/day	0.98 (0.76–1.27)
	Steady-heavy	1.61 (1.32–1.97)	≥50 g/day	1.43 (1.27–1.61)
**Gallbladder and biliary tract cancer**	Non-drinking	Reference	0 g/day	Reference
	Light	1.11 (0.98–1.26)	1–9.9 g/day	1.04 (0.92–1.17)
	Moderate	1.20 (1.07–1.35)	10–19.9 g/day	1.14 (0.99–1.32)
	Decreasing-heavy	1.09 (0.77–1.54)	20–29.9 g/day	1.38 (1.16–1.63)
	Increasing-heavy	1.36 (1.07–1.73)	30–49.9 g/day	1.38 (0.89–2.13)
	Steady-heavy	1.31 (0.87–1.97)	≥50 g/day	1.24 (0.99–1.56)
**Pancreatic cancer**	Non-drinking	Reference	0 g/day	Reference
	Light	1.11 (0.98–1.26)	1–9.9 g/day	1.02 (0.90–1.15)
	Moderate	1.04 (0.92–1.17)	10–19.9 g/day	0.97 (0.83–1.12)
	Decreasing-heavy	0.84 (0.56–1.26)	20–29.9 g/day	1.20 (1.00–1.44)
	Increasing-heavy	1.41 (1.11–1.79)	30–49.9 g/day	0.82 (0.47–1.42)
	Steady-heavy	1.48 (1.00–2.19)	≥50 g/day	0.97 (0.74–1.26)
**Laryngeal cancer**	Non-drinking	Reference	0 g/day	Reference
	Light	1.14 (0.84–1.53)	1–9.9 g/day	0.81 (0.61–1.08)
	Moderate	1.42 (1.09–1.86)	10–19.9 g/day	0.99 (0.71–1.39)
	Decreasing-heavy	1.91 (0.99–3.66)	20–29.9 g/day	1.53 (1.06–2.22)
	Increasing-heavy	1.80 (1.07–3.01)	30–49.9 g/day	1.31 (0.48–3.54)
	Steady-heavy	1.95 (0.86–4.46)	≥50 g/day	1.42 (0.87–2.31)
**Lung cancer**	Non-drinking	Reference	0 g/day	Reference
	Light	1.05 (0.98–1.12)	1–9.9 g/day	0.82 (0.76–0.88)
	Moderate	1.01 (0.95–1.08)	10–19.9 g/day	0.98 (0.90–1.06)
	Decreasing-heavy	0.94 (0.77–1.16)	20–29.9 g/day	0.99 (0.89–1.10)
	Increasing-heavy	1.39 (1.22–1.58)	30–49.9 g/day	1.33 (1.04–1.70)
	Steady-heavy	1.19 (0.94–1.50)	≥50 g/day	1.04 (0.91–1.19)
**Breast cancer**	Non-drinking	Reference	0 g/day	Reference
	Light	1.12 (0.57–2.18)	1–9.9 g/day	0.93 (0.50–1.72)
	Moderate	1.04 (0.56–1.93)	10–19.9 g/day	0.82 (0.37–1.81)
	Decreasing-heavy	2.20 (0.51–9.51)	20–29.9 g/day	1.69 (0.70–4.07)
	Increasing-heavy	2.23 (0.75–6.61)	30–49.9 g/day	-
	Steady-heavy	1.86 (0.25–13.96)	≥50 g/day	3.31 (1.36–8.03)
**Prostate cancer**	Non-drinking	Reference	0 g/day	Reference
	Light	1.04 (0.99–1.10)	1–9.9 g/day	1.02 (0.97–1.07)

	Moderate	1.05 (1.00–1.10)	10–19.9 g/day	1.07 (1.00–1.14)
	Decreasing-heavy	1.00 (0.86–1.16)	20–29.9 g/day	1.02 (0.94–1.11)
	Increasing-heavy	0.99 (0.88–1.11)	30–49.9 g/day	1.06 (0.86–1.30)
	Steady-heavy	1.06 (0.88–1.28)	≥50 g/day	0.97 (0.87–1.09)
**Testis cancer**	Non-drinking	Reference	0 g/day	Reference
	Light	0.77 (0.39–1.55)	1–9.9 g/day	1.93 (1.14–3.27)
	Moderate	1.39 (0.80–2.43)	10–19.9 g/day	1.05 (0.50–2.19)
	Decreasing-heavy	2.23 (0.52–9.61)	20–29.9 g/day	1.72 (0.66–4.50)
	Increasing-heavy	0.54 (0.07–4.04)	30–49.9 g/day	-
	Steady-heavy	-	≥50 g/day	2.20 (0.66–7.33)
**Kidney cancer**	Non-drinking	Reference	0 g/day	Reference
	Light	0.98 (0.86–1.10)	1–9.9 g/day	0.99 (0.88–1.10)
	Moderate	0.99 (0.88–1.10)	10–19.9 g/day	1.02 (0.90–1.16)
	Decreasing-heavy	1.18 (0.84–1.65)	20–29.9 g/day	0.90 (0.73–1.11)
	Increasing-heavy	0.97 (0.74–1.27)	30–49.9 g/day	1.26 (0.87–1.82)
	Steady-heavy	1.30 (0.85–1.97)	≥50 g/day	1.10 (0.86–1.42)
**Bladder cancer**	Non-drinking	Reference	0 g/day	Reference
	Light	1.02 (0.91–1.14)	1–9.9 g/day	0.91 (0.82–1.02)
	Moderate	1.04 (0.93–1.15)	10–19.9 g/day	0.96 (0.84–1.09)
	Decreasing-heavy	0.97 (0.71–1.34)	20–29.9 g/day	1.00 (0.85–1.19)
	Increasing-heavy	1.08 (0.86–1.36)	30–49.9 g/day	0.77 (0.47–1.26)
	Steady-heavy	0.95 (0.63–1.44)	≥50 g/day	0.95 (0.76–1.19)
**Brain cancer**	Non-drinking	Reference	0 g/day	Reference
	Light	0.96 (0.79–1.15)	1–9.9 g/day	0.94 (0.79–1.12)
	Moderate	0.78 (0.66–0.94)	10–19.9 g/day	0.85 (0.68–1.06)
	Decreasing-heavy	0.76 (0.40–1.43)	20–29.9 g/day	0.75 (0.53–1.06)
	Increasing-heavy	0.89 (0.58–1.36)	30–49.9 g/day	1.06 (0.55–2.05)
	Steady-heavy	1.18 (0.61–2.29)	≥50 g/day	0.90 (0.58–1.38)
**Thyroid cancer**	Non-drinking	Reference	0 g/day	Reference
	Light	0.93 (0.87–1.00)	1–9.9 g/day	0.99 (0.93–1.05)
	Moderate	0.92 (0.86–0.98)	10–19.9 g/day	0.92 (0.86–0.99)
	Decreasing-heavy	0.79 (0.60–1.04)	20–29.9 g/day	0.92 (0.81–1.05)
	Increasing-heavy	0.98 (0.83–1.16)	30–49.9 g/day	1.10 (0.88–1.38)
	Steady-heavy	0.45 (0.28–0.74)	≥50 g/day	0.71 (0.57–0.88)
**Hodgkin lymphoma**	Non-drinking	Reference	0 g/day	Reference
	Light	1.38 (0.59–3.21)	1–9.9 g/day	1.12 (0.50–2.49)
	Moderate	0.89 (0.38–2.10)	10–19.9 g/day	0.95 (0.34–2.64)
	Decreasing-heavy	-	20–29.9 g/day	1.59 (0.46–5.48)
	Increasing-heavy	-	30–49.9 g/day	-
	Steady-heavy	-	≥50 g/day	-

**Non-Hodgkin lymphoma**	Non-drinking	Reference	0 g/day	Reference
	Light	0.94 (0.82–1.07)	1–9.9 g/day	0.97 (0.85–1.10)
	Moderate	0.90 (0.79–1.02)	10–19.9 g/day	0.86 (0.73–1.01)
	Decreasing-heavy	0.76 (0.48–1.20)	20–29.9 g/day	0.66 (0.51–0.86)
	Increasing-heavy	0.81 (0.59–1.13)	30–49.9 g/day	1.06 (0.65–1.71)
	Steady-heavy	0.34 (0.14–0.82)	≥50 g/day	0.64 (0.45–0.92)
**Multiple myeloma and malignant plasma cell neoplasms**	Non-drinking	Reference	0 g/day	Reference
	Light	0.80 (0.64–1.02)	1–9.9 g/day	0.91 (0.73–1.13)
	Moderate	0.94 (0.76–1.15)	10–19.9 g/day	0.94 (0.73–1.23)
	Decreasing-heavy	0.98 (0.52–1.85)	20–29.9 g/day	1.16 (0.84–1.62)
	Increasing-heavy	0.89 (0.54–1.47)	30–49.9 g/day	0.18 (0.03–1.29)
	Steady-heavy	0.66 (0.25–1.78)	≥50 g/day	0.83 (0.50–1.38)
**Leukemia**	Non-drinking	Reference	0 g/day	Reference
	Light	0.85 (0.72–1.01)	1–9.9 g/day	0.90 (0.77–1.05)
	Moderate	0.85 (0.73–0.99)	10–19.9 g/day	0.78 (0.64–0.95)
	Decreasing-heavy	1.00 (0.61–1.63)	20–29.9 g/day	0.82 (0.62–1.10)
	Increasing-heavy	0.57 (0.36–0.90)	30–49.9 g/day	0.71 (0.35–1.43)
	Steady-heavy	1.22 (0.68–2.17)	≥50 g/day	1.14 (0.81–1.60)

aHR, adjusted hazard ratios; CI, confidence interval.

[a]Adjusted for age, income, body mass index, smoking status, physical activity, family history of cancer, and Charlson comorbidity index.

eTable 8. Adjusted hazard ratios[a] for the association between alcohol consumption trajectories and the cancer risk in case of excluding all cancer cases diagnosed within 1 year after the exposure measurement period (2002–2007) (N=2,821,315)

Cancer types	Alcohol drinking trajectories		Baseline alcohol drinking levels	
	**Groups**	**aHR (95% CI)**	**Groups**	**aHR (95% CI)**
**All cancers combined**	Non-drinking	Reference	0 g/day	Reference
	Light	1.03 (1.01–1.04)	1–9.9 g/day	0.98 (0.97–1.00)
	Moderate	1.06 (1.05–1.08)	10–19.9 g/day	1.03 (1.02–1.05)
	Decreasing-heavy	1.19 (1.15–1.22)	20–29.9 g/day	1.11 (1.09–1.13)
	Increasing-heavy	1.23 (1.20–1.26)	30–49.9 g/day	1.12 (1.08–1.16)
	Steady-heavy	1.35 (1.30–1.40)	≥50 g/day	1.24 (1.21–1.26)
**Alcohol-related cancers combined**	Non-drinking	Reference	0 g/day	Reference
	Light	1.09 (1.05–1.12)	1–9.9 g/day	1.02 (1.00–1.05)
	Moderate	1.22 (1.19–1.26)	10–19.9 g/day	1.16 (1.13–1.19)
	Decreasing-heavy	1.63 (1.54–1.72)	20–29.9 g/day	1.36 (1.31–1.40)
	Increasing-heavy	1.62 (1.55–1.68)	30–49.9 g/day	1.40 (1.31–1.49)
	Steady-heavy	1.86 (1.75–1.98)	≥50 g/day	1.65 (1.59–1.71)
**Lip, oral cavity, and pharyngeal cancer**	Non-drinking	Reference	0 g/day	Reference
	Light	1.14 (1.00–1.30)	1–9.9 g/day	1.07 (0.95–1.19)
	Moderate	1.33 (1.18–1.49)	10–19.9 g/day	1.27 (1.13–1.42)
	Decreasing-heavy	1.69 (1.35–2.12)	20–29.9 g/day	1.48 (1.29–1.70)
	Increasing-heavy	1.65 (1.38–1.98)	30–49.9 g/day	1.32 (1.00–1.73)
	Steady-heavy	2.12 (1.65–2.73)	≥50 g/day	1.78 (1.51–2.09)
**Esophageal cancer**	Non-drinking	Reference	0 g/day	Reference
	Light	1.65 (1.38–1.97)	1–9.9 g/day	1.38 (1.20–1.58)
	Moderate	3.26 (2.81–3.79)	10–19.9 g/day	2.07 (1.80–2.37)
	Decreasing-heavy	7.16 (5.85–8.76)	20–29.9 g/day	4.04 (3.54–4.63)
	Increasing-heavy	7.87 (6.61–9.37)	30–49.9 g/day	4.49 (3.55–5.68)
	Steady-heavy	8.49 (6.82–10.56)	≥50 g/day	5.69 (4.95–6.55)
**Gastric cancer**	Non-drinking	Reference	0 g/day	Reference
	Light	1.06 (1.03–1.10)	1–9.9 g/day	1.00 (0.97–1.03)
	Moderate	1.13 (1.10–1.17)	10–19.9 g/day	1.10 (1.06–1.13)
	Decreasing-heavy	1.24 (1.16–1.33)	20–29.9 g/day	1.15 (1.11–1.20)
	Increasing-heavy	1.35 (1.28–1.42)	30–49.9 g/day	1.17 (1.08–1.26)
	Steady-heavy	1.39 (1.28–1.50)	≥50 g/day	1.25 (1.19–1.31)
**Colorectal cancer**	Non-drinking	Reference	0 g/day	Reference
	Light	1.07 (1.03–1.12)	1–9.9 g/day	1.08 (1.04–1.12)
	Moderate	1.27 (1.22–1.31)	10–19.9 g/day	1.22 (1.18–1.27)

	Decreasing-heavy	1.47 (1.36–1.59)	20–29.9 g/day	1.30 (1.24–1.36)
	Increasing-heavy	1.43 (1.35–1.52)	30–49.9 g/day	1.37 (1.26–1.50)
	Steady-heavy	1.59 (1.45–1.74)	≥50 g/day	1.44 (1.37–1.53)
**Liver cancer**	Non-drinking	Reference	0 g/day	Reference
	Light	1.04 (0.99–1.09)	1–9.9 g/day	0.90 (0.86–0.94)
	Moderate	0.99 (0.94–1.03)	10–19.9 g/day	0.97 (0.92–1.01)
	Decreasing-heavy	1.40 (1.28–1.54)	20–29.9 g/day	1.16 (1.09–1.23)
	Increasing-heavy	1.40 (1.30–1.51)	30–49.9 g/day	1.17 (1.04–1.31)
	Steady-heavy	1.69 (1.52–1.88)	≥50 g/day	1.50 (1.41–1.61)
**Gallbladder and biliary tract cancer**	Non-drinking	Reference	0 g/day	Reference
	Light	1.14 (1.04–1.26)	1–9.9 g/day	1.05 (0.96–1.14)
	Moderate	1.18 (1.08–1.29)	10–19.9 g/day	1.07 (0.97–1.18)
	Decreasing-heavy	1.36 (1.12–1.64)	20–29.9 g/day	1.31 (1.17–1.46)
	Increasing-heavy	1.43 (1.24–1.66)	30–49.9 g/day	1.19 (0.93–1.53)
	Steady-heavy	1.54 (1.24–1.92)	≥50 g/day	1.51 (1.33–1.72)
**Pancreatic cancer**	Non-drinking	Reference	0 g/day	Reference
	Light	1.03 (0.94–1.13)	1–9.9 g/day	0.97 (0.89–1.05)
	Moderate	1.03 (0.95–1.12)	10–19.9 g/day	1.05 (0.97–1.15)
	Decreasing-heavy	1.08 (0.90–1.31)	20–29.9 g/day	1.05 (0.94–1.17)
	Increasing-heavy	1.25 (1.09–1.44)	30–49.9 g/day	1.22 (0.98–1.50)
	Steady-heavy	1.39 (1.13–1.71)	≥50 g/day	1.12 (0.98–1.28)
**Laryngeal cancer**	Non-drinking	Reference	0 g/day	Reference
	Light	1.26 (1.05–1.52)	1–9.9 g/day	0.95 (0.81–1.11)
	Moderate	1.50 (1.28–1.76)	10–19.9 g/day	1.24 (1.06–1.46)
	Decreasing-heavy	2.42 (1.85–3.17)	20–29.9 g/day	1.63 (1.37–1.95)
	Increasing-heavy	2.43 (1.94–3.03)	30–49.9 g/day	1.92 (1.39–2.65)
	Steady-heavy	2.78 (2.05–3.75)	≥50 g/day	2.10 (1.74–2.55)
**Lung cancer**	Non-drinking	Reference	0 g/day	Reference
	Light	0.94 (0.90–0.98)	1–9.9 g/day	0.85 (0.82–0.88)
	Moderate	0.94 (0.90–0.97)	10–19.9 g/day	0.91 (0.87–0.95)
	Decreasing-heavy	1.03 (0.95–1.12)	20–29.9 g/day	0.97 (0.93–1.02)
	Increasing-heavy	1.14 (1.07–1.22)	30–49.9 g/day	1.10 (1.00–1.22)
	Steady-heavy	1.22 (1.12–1.34)	≥50 g/day	1.15 (1.08–1.21)
**Breast cancer**	Non-drinking	Reference	0 g/day	Reference
	Light	1.46 (0.83–2.57)	1–9.9 g/day	1.11 (0.68–1.80)
	Moderate	1.54 (0.92–2.58)	10–19.9 g/day	1.08 (0.63–1.86)
	Decreasing-heavy	2.01 (0.69–5.91)	20–29.9 g/day	1.82 (0.99–3.37)
	Increasing-heavy	1.07 (0.36–3.13)	30–49.9 g/day	1.70 (0.52–5.59)
	Steady-heavy	3.23 (1.10–9.50)	≥50 g/day	2.99 (1.57–5.72)
	Non-drinking	Reference	0 g/day	Reference

**Prostate cancer**	Light	1.06 (1.01–1.10)	1–9.9 g/day	1.03 (0.99–1.07)
	Moderate	1.04 (1.01–1.08)	10–19.9 g/day	1.05 (1.00–1.10)
	Decreasing-heavy	1.03 (0.93–1.13)	20–29.9 g/day	1.02 (0.96–1.07)
	Increasing-heavy	1.03 (0.96–1.11)	30–49.9 g/day	1.06 (0.94–1.20)
	Steady-heavy	1.11 (0.99–1.24)	≥50 g/day	1.03 (0.96–1.10)
**Testis cancer**	Non-drinking	Reference	0 g/day	Reference
	Light	1.16 (0.73–1.83)	1–9.9 g/day	1.59 (1.09–2.32)
	Moderate	1.23 (0.82–1.86)	10–19.9 g/day	1.23 (0.81–1.87)
	Decreasing-heavy	1.22 (0.47–3.15)	20–29.9 g/day	1.75 (1.05–2.93)
	Increasing-heavy	0.49 (0.17–1.39)	30–49.9 g/day	0.28 (0.04–2.03)
	Steady-heavy	1.30 (0.39–4.27)	≥50 g/day	1.42 (0.68–2.93)
**Kidney cancer**	Non-drinking	Reference	0 g/day	Reference
	Light	1.00 (0.91–1.10)	1–9.9 g/day	1.02 (0.94–1.10)
	Moderate	1.05 (0.97–1.14)	10–19.9 g/day	1.07 (0.99–1.16)
	Decreasing-heavy	1.03 (0.84–1.26)	20–29.9 g/day	1.05 (0.94–1.18)
	Increasing-heavy	1.04 (0.89–1.21)	30–49.9 g/day	1.09 (0.89–1.32)
	Steady-heavy	1.13 (0.89–1.44)	≥50 g/day	1.01 (0.87–1.18)
**Bladder cancer**	Non-drinking	Reference	0 g/day	Reference
	Light	1.02 (0.94–1.10)	1–9.9 g/day	0.93 (0.87–1.00)
	Moderate	1.00 (0.93–1.07)	10–19.9 g/day	0.96 (0.89–1.04)
	Decreasing-heavy	0.96 (0.81–1.13)	20–29.9 g/day	0.98 (0.89–1.08)
	Increasing-heavy	1.03 (0.90–1.17)	30–49.9 g/day	0.84 (0.67–1.04)
	Steady-heavy	1.09 (0.90–1.33)	≥50 g/day	0.98 (0.87–1.10)
**Brain cancer**	Non-drinking	Reference	0 g/day	Reference
	Light	0.92 (0.80–1.06)	1–9.9 g/day	0.86 (0.76–0.97)
	Moderate	0.86 (0.76–0.97)	10–19.9 g/day	0.94 (0.82–1.07)
	Decreasing-heavy	0.65 (0.46–0.93)	20–29.9 g/day	0.84 (0.70–1.01)
	Increasing-heavy	0.81 (0.64–1.04)	30–49.9 g/day	1.13 (0.83–1.53)
	Steady-heavy	0.97 (0.67–1.40)	≥50 g/day	0.76 (0.59–0.97)
**Thyroid cancer**	Non-drinking	Reference	0 g/day	Reference
	Light	0.96 (0.91–1.02)	1–9.9 g/day	1.00 (0.95–1.04)
	Moderate	0.93 (0.88–0.97)	10–19.9 g/day	0.91 (0.86–0.95)
	Decreasing-heavy	0.78 (0.67–0.89)	20–29.9 g/day	0.90 (0.83–0.96)
	Increasing-heavy	0.89 (0.81–0.98)	30–49.9 g/day	0.96 (0.86–1.08)
	Steady-heavy	0.82 (0.68–0.98)	≥50 g/day	0.83 (0.75–0.93)
**Hodgkin lymphoma**	Non-drinking	Reference	0 g/day	Reference
	Light	0.99 (0.60–1.65)	1–9.9 g/day	1.14 (0.75–1.73)
	Moderate	0.91 (0.58–1.43)	10–19.9 g/day	0.74 (0.45–1.20)
	Decreasing-heavy	0.46 (0.11–1.95)	20–29.9 g/day	1.06 (0.59–1.90)
	Increasing-heavy	0.48 (0.17–1.37)	30–49.9 g/day	0.26 (0.04–1.90)

	Steady-heavy	0.76 (0.18–3.20)	≥50 g/day	0.38 (0.12–1.22)
**Non-Hodgkin lymphoma**	Non-drinking	Reference	0 g/day	Reference
	Light	0.93 (0.83–1.03)	1–9.9 g/day	0.94 (0.86–1.03)
	Moderate	0.88 (0.81–0.97)	10–19.9 g/day	0.85 (0.77–0.94)
	Decreasing-heavy	0.92 (0.73–1.17)	20–29.9 g/day	0.88 (0.77–1.01)
	Increasing-heavy	0.90 (0.75–1.08)	30–49.9 g/day	1.01 (0.79–1.29)
	Steady-heavy	0.85 (0.63–1.16)	≥50 g/day	0.93 (0.78–1.11)
**Multiple myeloma and malignant plasma cell neoplasms**	Non-drinking	Reference	0 g/day	Reference
	Light	0.78 (0.66–0.94)	1–9.9 g/day	0.89 (0.76–1.04)
	Moderate	0.85 (0.73–0.99)	10–19.9 g/day	0.86 (0.73–1.02)
	Decreasing-heavy	0.97 (0.67–1.40)	20–29.9 g/day	0.81 (0.65–1.02)
	Increasing-heavy	0.90 (0.67–1.20)	30–49.9 g/day	0.59 (0.34–1.02)
	Steady-heavy	0.77 (0.46–1.27)	≥50 g/day	0.92 (0.70–1.21)
**Leukemia**	Non-drinking	Reference	0 g/day	Reference
	Light	0.93 (0.82–1.06)	1–9.9 g/day	0.94 (0.85–1.05)
	Moderate	0.86 (0.77–0.96)	10–19.9 g/day	0.80 (0.71–0.90)
	Decreasing-heavy	0.88 (0.67–1.17)	20–29.9 g/day	0.93 (0.79–1.09)
	Increasing-heavy	0.82 (0.66–1.03)	30–49.9 g/day	0.70 (0.50–0.99)
	Steady-heavy	1.04 (0.75–1.44)	≥50 g/day	1.02 (0.84–1.24)

aHR, adjusted hazard ratios; CI, confidence interval.

[a]Adjusted for age, income, body mass index, smoking status, physical activity, family history of cancer, and Charlson comorbidity index.

eTable 9. Adjusted hazard ratios[a] (95% confidence interval) for the association between alcohol consumption trajectories and the cancer risk with the combination of the three alcohol trajectories into one group

Cancer types	Alcohol drinking trajectories	aHR (95% CI)
**All cancers combined**	Non-drinking	Reference
	Light	1.03 (1.02–1.05)
	Moderate	1.06 (1.05–1.08)
	Heavy	1.24 (1.21–1.26)
**Alcohol-related cancers combined**	Non-drinking	Reference
	Light	1.09 (1.06–1.12)
	Moderate	1.21 (1.18–1.24)
	Heavy	1.64 (1.59–1.69)
**Lip, oral cavity, and pharyngeal cancer**	Non-drinking	Reference
	Light	1.18 (1.05–1.33)
	Moderate	1.32 (1.18–1.46)
	Heavy	1.75 (1.53–2.00)
**Esophageal cancer**	Non-drinking	Reference
	Light	1.64 (1.39–1.95)
	Moderate	3.21 (2.78–3.70)
	Heavy	7.75 (6.67–9.00)
**Gastric cancer**	Non-drinking	Reference
	Light	1.07 (1.03–1.10)
	Moderate	1.14 (1.11–1.17)
	Heavy	1.33 (1.28–1.38)
**Colorectal cancer**	Non-drinking	Reference
	Light	1.08 (1.04–1.12)
	Moderate	1.27 (1.23–1.32)
	Heavy	1.46 (1.40–1.53)
**Liver cancer**	Non-drinking	Reference
	Light	1.03 (0.98–1.08)
	Moderate	0.96 (0.92–1.00)
	Heavy	1.40 (1.32–1.48)
**Gallbladder and biliary tract cancer**	Non-drinking	Reference
	Light	1.15 (1.05–1.26)
	Moderate	1.20 (1.10–1.30)
	Heavy	1.49 (1.34–1.67)
**Pancreatic cancer**	Non-drinking	Reference
	Light	1.04 (0.96–1.14)
	Moderate	1.03 (0.95–1.12)
	Heavy	1.21 (1.08–1.34)

**Laryngeal cancer**	Non-drinking	Reference
	Light	1.21 (1.02–1.44)
	Moderate	1.48 (1.27–1.72)
	Heavy	2.49 (2.09–2.96)
**Lung cancer**	Non-drinking	Reference
	Light	0.94 (0.90–0.98)
	Moderate	0.94 (0.91–0.97)
	Heavy	1.12 (1.07–1.18)
**Breast cancer**	Non-drinking	Reference
	Light	1.30 (0.76–2.21)
	Moderate	1.33 (0.82–2.15)
	Heavy	1.41 (0.70–2.83)
**Prostate cancer**	Non-drinking	Reference
	Light	1.05 (1.01–1.10)
	Moderate	1.04 (1.00–1.07)
	Heavy	1.04 (0.98–1.10)
**Testis cancer**	Non-drinking	Reference
	Light	1.23 (0.79–1.90)
	Moderate	1.25 (0.84–1.85)
	Heavy	0.95 (0.51–1.76)
**Kidney cancer**	Non-drinking	Reference
	Light	1.00 (0.92–1.10)
	Moderate	1.05 (0.98–1.14)
	Heavy	1.04 (0.93–1.17)
**Bladder cancer**	Non-drinking	Reference
	Light	1.03 (0.96–1.11)
	Moderate	1.00 (0.94–1.07)
	Heavy	1.02 (0.93–1.12)
**Brain cancer**	Non-drinking	Reference
	Light	0.95 (0.83–1.08)
	Moderate	0.86 (0.76–0.97)
	Heavy	0.84 (0.70–1.00)
**Thyroid cancer**	Non-drinking	Reference
	Light	0.97 (0.92–1.02)
	Moderate	0.93 (0.88–0.97)
	Heavy	0.83 (0.77–0.90)
**Hodgkin lymphoma**	Non-drinking	Reference
	Light	1.03 (0.62–1.71)
	Moderate	0.95 (0.61–1.49)
	Heavy	0.52 (0.24–1.15)
**Non-Hodgkin lymphoma**	Non-drinking	Reference
	Light	0.94 (0.85–1.04)

	Moderate	0.89 (0.81–0.97)
	Heavy	0.91 (0.79–1.04)
**Multiple myeloma and malignant plasma cell neoplasms**	Non-drinking	Reference
	Light	0.79 (0.66–0.93)
	Moderate	0.85 (0.74–0.99)
	Heavy	0.87 (0.70–1.09)
**Leukemia**	Non-drinking	Reference
	Light	0.92 (0.81–1.03)
	Moderate	0.85 (0.77–0.95)
	Heavy	0.87 (0.74–1.03)

aHR, adjusted hazard ratios; CI, confidence interval.

[a]Adjusted for age, income, body mass index, smoking status, physical activity, family history of cancer, and Charlson comorbidity index.
