# Supplementary material for: Erratum for “Cancer Risk According to Alcohol Consumption Trajectories: A Population-based Cohort Study of 2.8 Million Korean Men”
Source: J Epidemiol. 2023 Dec 5;33(12):649–51. doi: 10.2188/jea.JE20230158 (PMC10635815; doi:10.2188/jea.JE20230158)
Supplement: Supplementary file 1 [file je-33-649-s001.pdf]

eTable 1. Erratum in the main text

Before correction	After correction
The risk increased by 7–40% for gastric cancer and 15–61% for gallbladder and biliary tract cancers **following** light intake (page 4, 13th line of the 2nd paragraph).	The risk increased by 7–40% for gastric cancer and 15–61% for gallbladder and biliary tract cancers **from** light intake.
A significant inverse association was observed for thyroid cancer **following** moderate intake (page 4, 17th line of the 2nd paragraph).	A significant inverse association was observed for thyroid cancer **from** moderate intake.
In particular, the risk for all cancers combined in the TR4 (decreasing-heavy) trajectory significantly decreased by 11–12% (page 4, 4th line of the 3rd paragraph).	In particular, the risk for all cancers **and all alcohol-related cancers** combined in the TR4 (decreasing-heavy) trajectory significantly decreased by 11–12%.
They were then classified into three trajectories: TR2 (light; 32.7%), TR3 (moderate; 66.2%), and **TR4** (increasing-heavy; 1.1%) (page 7, 13th line of the 1st paragraph).	They were then classified into three trajectories: TR2 (light; 32.7%), TR3 (moderate; 66.2%), and **TR5** (increasing-heavy; 1.1%).
